# Spiders on a Hot Volcanic Roof: Colonisation Pathways and Phylogeography of the Canary Islands Endemic Trap-Door Spider *Titanidiops canariensis* (Araneae, Idiopidae)

**DOI:** 10.1371/journal.pone.0115078

**Published:** 2014-12-10

**Authors:** Vera Opatova, Miquel A. Arnedo

**Affiliations:** Institut de Recerca de la Biodiversitat & Departament de Biologia Animal, Universitat de Barcelona, Barcelona, Spain; University of Lausanne, Switzerland

## Abstract

Studies conducted on volcanic islands have greatly contributed to our current understanding of how organisms diversify. The Canary Islands archipelago, located northwest of the coast of northern Africa, harbours a large number of endemic taxa. Because of their low vagility, mygalomorph spiders are usually absent from oceanic islands. The spider *Titanidiops canariensis*, which inhabits the easternmost islands of the archipelago, constitutes an exception to this rule. Here, we use a multi-locus approach that combines three mitochondrial and four nuclear genes to investigate the origins and phylogeography of this remarkable trap-door spider. We provide a timeframe for the colonisation of the Canary Islands using two alternative approaches: concatenation and species tree inference in a Bayesian relaxed clock framework. Additionally, we investigate the existence of cryptic species on the islands by means of a Bayesian multi-locus species delimitation method. Our results indicate that *T. canariensis* colonised the Canary Islands once, most likely during the Miocene, although discrepancies between the timeframes from different approaches make the exact timing uncertain. A complex evolutionary history for the species in the archipelago is revealed, which involves two independent colonisations of Fuerteventura from the ancestral range of *T. canariensis* in northern Lanzarote and a possible back colonisation of southern Lanzarote. The data further corroborate a previously proposed volcanic refugium, highlighting the impact of the dynamic volcanic history of the island on the phylogeographic patterns of the endemic taxa. *T. canariensis* includes at least two different species, one inhabiting the Jandia peninsula and central Fuerteventura and one spanning from central Fuerteventura to Lanzarote. Our data suggest that the extant northern African *Titanidiops* lineages may have expanded to the region after the islands were colonised and, hence, are not the source of colonisation. In addition, *T. maroccanus* may harbour several cryptic species.

## Introduction

Oceanic islands due to their volcanic origin are ideal systems for evolutionary studies [1]. Episodes of volcanic activity have left their fingerprints on the genetic diversity and distribution of endemic terrestrial organisms. Recurrent range shifts, geographic isolation and population bottlenecks driven by lava flows have shaped the complex phylogeographic patterns and have led speciation in local organisms [2], [3], [4], [5], [6], [7], [8], [9].

**Figure 1:**
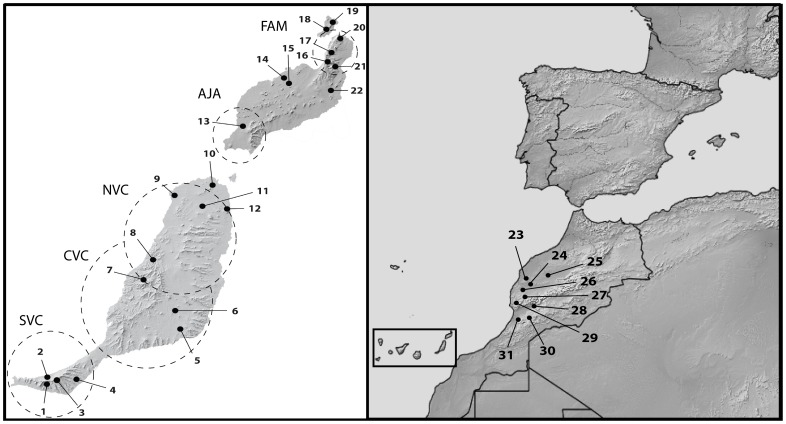
*Titanidiops* sampling locations, 1: Barranco del Ciervo, 2: Cofete, 3: Pico de Fraile, 4: Barranco Mal Nombre, 5: Tequital, 6: Caldera la Laguna, 7: Betancuria, 8: Valle de Aguas Verdes, 9: Faro Tscón, 10: Corralejo, 11: Villaverde, 12: Caldería de la Roja, 13: Salinas de Janubio, 14: Tinajo, 15: Tinache, 16: Valle de Malpaso, 17: Valle de Guinate, 18: Montaña de Mojón, 19: El Vallichuelo, 20: Mirador del Río, 21: Barranco Hondo del Valle, 22: Tejía, 23: Smi-Mou, 24: Kemis-oulat-el-Hadj, 25: Ouzoud Falls rd., 26: Jbele Amsittene, 27: Tamanat - Aid-Beoude rd., 28: Iguer rd., 29: Imoza rd. nr. Tourarin, 30: Ait-Aisa, 31: Aid-Baha. For detailed information, see S1 Table. The map was created using SimpleMappr http://www.simplemappr.net/. Circled areas correspond to the location of the Volcanic Complexes on Fuerteventura and Lanzarote [75]. SVC: Southern Volcanic Complex, CVC: Central Volcanic Complex, NVC: Northern Volcanic Complex, AJA: Los Ajaches, FAM: Famara.

The Canary Islands archipelago lies in the Atlantic Ocean, approximately 110 km from the north-western coast of Africa, comprising seven main islands and several smaller islets (Fig. 1). The region harbours a significant number of endemic organisms; 50% of the known invertebrates and 27% of the vascular plants inhabiting the archipelago are Canarian endemics. This extraordinary biological richness has been traditionally interpreted in many organisms as a relict of the Tertiary Mediterranean diversity, but the advent of molecular phylogenetics revealed a large amount of in situ diversification [10]. Some groups, however, have colonised the archipelago repeatedly [11], [12], [13], [14].

The archipelago was built by several cycles of volcanic activity tracing back to the Miocene and persisting until today [15], [16], [17]. The islands on the eastern side were the first to emerge, and the remaining islands appeared subsequently, following an east to west pattern [15]. Fuerteventura, Lanzarote and the surrounding islets, hereafter referred to as the Eastern Canary Islands, are the emergent parts of a volcanic ridge that runs parallel to the African coast. The islands are separated by shallow waters and have been repeatedly connected during marine transgressions [18]. The sub-aerial stage of Fuerteventura began approximately 22 million years ago (Ma), and the volcanism progressed in a SSW-NNE direction. After several rounds of volcanic activity followed by periods of erosional quiescence, post-erosional volcanic activity began in Lanzarote approximately 1.6 Ma, and eruptions have been documented even in historic times [18], [19].

The infraorder Mygalomorphae is one of the three main lineages recognised within spiders [20], representing approximately 6.3% of extant spider diversity [21]. Mygalomorphs are generally robust spiders that lack the ability to spin complicated web structures and present other characters regarded as plesiomorphic among the spiders, such as four book lungs and chelicerae with unsynchronised movement of the longitudinal fangs [22]. Mygalomorphs show high levels of local endemism, which is generally attributed to their low dispersal ability [23], [24]. They show phenological and behavioural sex dimorphism; females tend to be long-lived and sedentary, while short-lived adult males actively search for mates after moulting and, thus, mediate gene flow among the populations.

The taxonomy of the group is challenging, as most of the closely related taxa are morphologically homogenous and the majority of diagnostic characters are found in the reproductive organs of adult males, usually present in the field for a short period of time. Because of their uniform morphology and poor dispersal ability, which causes deep genetic structuring even among geographically close populations, mygalomorphs have become a model system to test species boundaries [24], [25], [26], [27].

**Figure 2:**
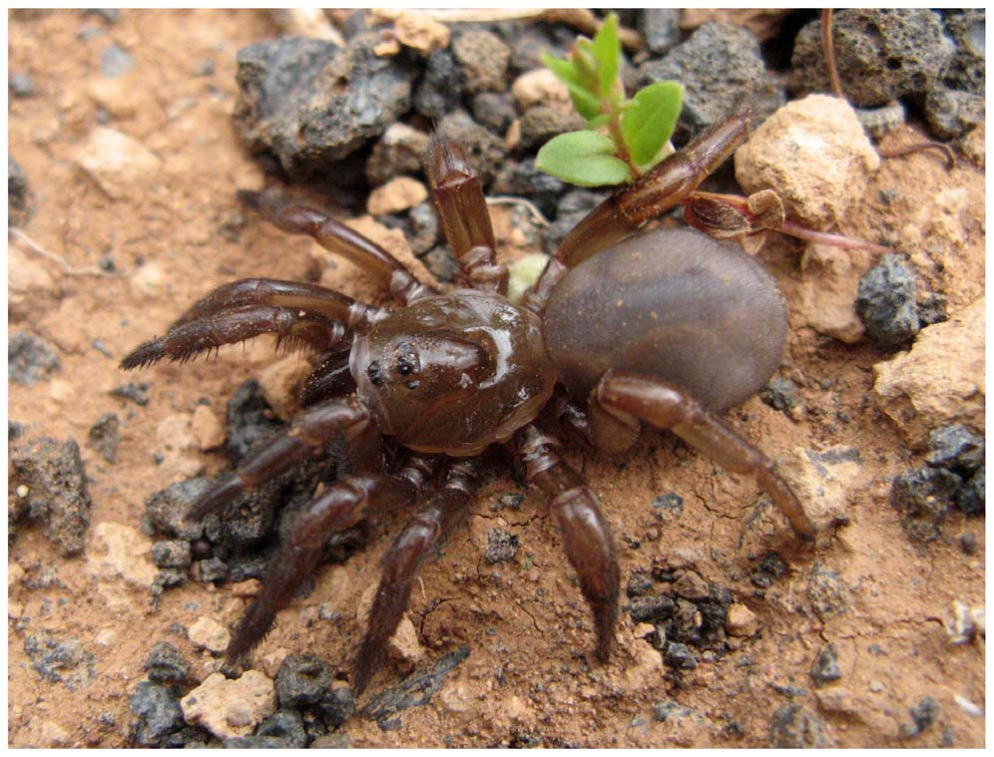
Picture of *Titanidiops canariensis* from loc. 7: Betancuria, photo credit VO.

Mygalomorphs are notoriously absent from oceanic islands, and the few exceptions involve species either in the Caribbean [21], belonging to groups for which airborne dispersal has been reported [28], [29], [30], or in the Australian region, where the existence of land bridges during sea level changes cannot be ruled out [31]. In this regard, the presence of the trap-door spider *Titanidiops canariensis* Wunderlich, 1992 (Fig. 2) on the Canary Islands has great biogeographic relevance, as the archipelago was never connected to the continent, and the family Idiopidae, to which it belongs, has had no reported cases of aerial dispersal.

Idiopids are widespread across Australia, New Zealand, South-East Asia, Sub-Saharan Africa, Madagascar and South America but also include a few disjoint species in North Africa and the Middle East [21]. This wide distribution is often attributed to the Gondwanan origin of the family [20], [32]. The family is well defined on a morphological basis [22], and its monophyly has been subsequently supported by molecular studies [20], [32], [33]. The genus *Titanidiops* currently comprises only three species: *T. canariensis*, endemic to the Canary Islands; *T. maroccanus* Simon, 1909 from Morocco; and the type species *T. compactus* Gerstäcker, 1873, which occurs in East Africa. *T. canariensis* inhabits the Eastern Canary Islands, where it can be found in most habitats with the exception of sand dunes and barren lava badlands (i.e., lava flows of recent origin). The spiders are mid-size and ground dwelling, and they construct underground, silk-lined burrows that open to the surface with a trap-door [34], [35]. There is almost no information known regarding its ecology, life cycle or phylogenetic affinities.

This study aims to uncover the phylogenetic origins of *T. canariensis*, one of the few examples of trap-door spiders endemic to an oceanic archipelago, and to infer the temporal framework for the colonisation of the islands using a multi-locus approach. Because of the low vagility of trap-door spiders and the dynamic volcanic history of the Eastern Canaries, we hypothesise deep and complex phylogeographic patterns, which may have led to the formation of cryptic species.

## Materials and Methods

### Taxonomic sampling

Most of the *Titanidiops* samples used in the present study were collected by the first author between 2009 and 2010. Additional specimens of *Titanidiops canariensis* and one specimen of the genus *Segregara* were kindly donated by colleagues. Three other representatives of the family Idiopidae were included in the analyses: *Idiops syriacus* from Israel, *Idiops* sp. from South Africa [20] and *Segregara* sp. from South Africa. The last species was used as an outgroup to root the trees. Detailed locality data are included in S1 Table. Sampling in the Eastern Canaries was conducted under permit num. 4012 granted by the Cabildo de Fuerteventura. Sampling in Morocco was conducted outside designated protected areas. The specimens of *Idiops syriacus* were collected under the collection permit 2011/38207 granted by the Israeli government to Y. Lubin and extended to the 26th European Congress of Arachnology participants.

### DNA extraction, PCR amplification, cloning and sequencing

Whole genomic DNA was extracted from the samples using the SpeedTools Tissue Extraction Kit (Biotools) following the manufacturer’s guidelines. Partial fragments of four mitochondrial and four nuclear genes were sequenced in present study: 5’ half of the Cytochrome oxidase I (*cox1*) (the animal barcode), the 3’ half of the 16 s rDNA (*16S*), the *tRNA-Leu* (*L1*) and the 5’ half of the NADH dehydrogenase subunit I (*nad1*), a fragment of the 28S rDNA (*28S*), Elongation factor-1 gamma (*EF1γ*), Histone H3 (*H3*) and one anonymous locus amplified with primers designed for the Heat Shock Protein Hsp 70 [36] (*AL1*-*Hsp70*), respectively.

The PCR amplifications were carried out with the following primer combinations. *Cox1* with the primer pair C1-J-1490/C1-N-2198 [37], the fragment comprising the 16S, L1 and *nad1* with the primer pair LR-N-13398 [38]/and N1-J-12261 [39] or, alternatively, only the 3’ half of the 16S with LR-N-13398 combined with LR-J-12864 [4]. All mitochondrial fragments were successfully amplified at annealing temperature range of 43–46°C. For the nuclear genes, *28S* was amplified with 28S-O/28S-B or 28S-O/28S-C [40], [41] at 62–64°C annealing temperature. The *EF1γ* fragment was amplified with primers ER1gF78/EF1γR1258 [32] and primer pair designed in the present study EF-gIDIf 5′- GGCAACAACCAGCTCGTGGA -3′/EF-gIDIr 5′- GTGCTGTTATTATCTTCGCC -3′, at 52°C annealing temperature. Histone *H3* was amplified with the primer combination H3a F/H3a R [42]. The *AL1*-*Hsp70* anonymous locus was amplified with the Heat Shock Protein primers MT70-MF3/MT70-R4 [36] and complemented with a newly designed primer pair for *T. canariensis* Hsp70-TitMF3 5′- AGCGACATGATGCCGAGAGT -3′/Hsp70-TitR4 5′- GGAGGATGCAGTGGACATGG -3’ yielding amplifications at 52–54°C. Some individuals showed *AL1*-*Hsp70* amplicons of different length and were further purified and cloned using the pGem-T kit (Promega).

All the reactions were carried out mixing 1.25 U *Taq* polymerase (Promega), 2.5 mM MgCl_2_ (Promega), 0.2 mM of each dNTP, 0.2 µM of each primer and 1.5 µl of DNA and the 5 µl of *Taq* buffer and adding ultrapure, distilled water up to a total reaction volume of 25 µl. PCR products were purified using ExoSAP-IT (USB Corporation) and sequenced in both directions using BigDye Terminator v3.1 Cycle Sequencing Kit (Applied Biosystems) on ABI 3700 automated sequencer at *Centres Científics i Tecnològics* of the University of Barcelona (CCiTUB, www.ccit.ub.edu) Spain. The chromatograms were assembled and edited in Geneious v. 5.3.6. [43].

### Sequence alignment, allele phasing and recombination testing

All gene fragments with exception of *nad1* and *H3* presented length polymorphism due to indel mutations. Sequence alignments of *28S*, *16S, L1* and *AL1*-*Hsp70* were obtained with the online version of MAFFT v. 6 [44], (available at http://mafft.cbrc.jp/alignment/server/) using the Q-INS-i approach with default settings (gap opening penalty GOP = 1.53 and offset value set to 0.0). Online version of the alignment program TranslatorX [45] (available at http://www.translatorx.co.uk/), which uses amino acid back translation to guide the nucleotide alignment, was used to build the alignments of protein coding genes *cox1* and *EF1γ*. The alignments of *nad1* and *H3* were trivial since no length variation was observed in this gene fragments.

The allelic phases of the nuclear gene fragments were determined using the PHASE algorithm [46], [47] as implemented in DnaSP 5.10.1 [48]. Recombination was tested by means of the difference of sum of squares method (DSS) as implemented in TOPALi v 2.5 [49], [50]. The size of sliding window was set to 500 bp in all fragments except for *H3*, where a 300 bp frame was used instead.

### Delimitation of putative independent evolutionary lineages

Single-locus genes provide useful information to generate preliminary hypotheses of species delineation [51]. Evolutionary processes such as introgression and incomplete lineage sorting and biological features such as dispersal ability and population sizes, however, may compromise the ability of single markers to infer species boundaries [52], [53], [54]. Therefore candidate lineages have to be subsequently validated by using multi-locus, multi-coalescent approaches, and further corroborated by the addition of phenotypic/ecological information [55].

The General Mixed Yule Coalescence model (GMYC) [56] was used to delimit coalescent groups (i.e. putative independent evolutionary lineages) within the complete *Titanidiops cox1* sequence data set due to the consistent amplification of this fragment across the individuals and its higher variability. The computer program BEAST was used to infer an ultrameric tree for the whole *cox1* data set defining a lognormal relaxed clock, with a single partition, under a GTR+Γ evolutionary model, the ucld.mean parameter set to 1 and selecting a constant population size coalescent tree prior [57]. Two independent runs of 5×10^7^ generations were conducted remotely at the BIOPORTAL computer resource of the University of Oslo (http://www.bioportal.uio.no/). Convergence between runs and correct mixing within each run were visualized with TRACER [58]. Individual runs were combined in BEAST accompanying program LOGCOMBINER. The first 10% of the generations of each run was discarded as a burn-in. A consensus chronogram was inferred with TREEANNOTATOR. The GMYC analysis was carried out in the R (http://www.r-project.org) environment using the SPLITS package [59].

### Phylogenetic analysis

Phylogenetic inference was conducted under two different approaches: (1) by assuming a common underlying tree for the different genes (i.e. concatenation approach) and (2) by assuming independent gene trees and species tree (i.e. species tree/gene tree approach). The *cox1*, *16S*, *L1*, *nad1*, *28S*, *EF1γ, H3* gene fragments of a single individual with the most complete sequences from each *Titanidiops canariensis* GMYC cluster identified above were concatenated in a single matrix using Geneious v. 5.3.6. [43]. The gaps were coded as presence/absence characters following the simple coding approach [60] as implemented in FastGap 1.2 [61], (available at http://www.aubot.dk/FastGap_home.htm). The *AL1*-*Hsp70* gene fragment was not included in the concatenated matrix because of the deep differences observed within individual alleles and the absence of data for Moroccan samples.

The best partitioning scheme and evolutionary model were selected using the greedy algorithm in the program PARTITIONFINDER [62]. The *L1* and *16S* genes were combined in a single partition.

The Bayesian inference analyses were conducted in MrBayes v. 3.1.2 [63] and run remotely at the CIPRES portal [64]. A ten-partition scheme with the corresponding evolutionary models as selected by PARTITIONFINDER and an additional restriction model for binary scored gaps were defined (see Table 1). Two independent runs of 5×10^7^ generations with 8 MCMC (Markov Chain Monte Carlo) chains each, starting from random trees and resampling each 1000 generations were run simultaneously. The first 20% of the generations were discarded as a *burn-in* for the analyses. Convergence of the runs was assessed by monitoring the standard deviation of split frequencies (<0.01) with the help of the program TRACER v.1.5 [58], which was further used to assess correct mixing within each chain.

**Table 1:** Evolutionary models and partition schemes selected by PARTITIONFINDER for the different analyses conducted.

Concatenated mtDNA + nucDNA		Concatenated mtDNA time		Coalescent mtDNA + nucDNA	
Partition	Model	Partition	Model	Partition	Model
*cox1* 1^st^	HKY+I+G	*cox1*	GTR+I+G	mtDNA	GTR+G
*cox1*, *nad1* 2^nd^	K81+I+G	*16S-L1*	GTR+I+G	*28S*	HKY+I
*cox1* 3^rd^	K81+G	*nad1*	GTR+I+G	*EF1γ*	HKY+I+G
*nad1* 3^rd^	HKY+G			*H3*	K80+I
*nad* 1^st^, *16SL1*, *H3* 3^rd^	GTR+I+G			*AL1*-*Hsp70*	K80+G
*EF1γ* 1^st^	JC+I				
*EF1γ* 2^nd^	F81+I				
*EF1γ* 3^rd^	K80+G				
*28S*	HKY+I				
H3 2^nd^, 3^rd^	K80+I				

Concatenated mtDNA time refers to concatenated analyses conducted with BEAST including informed substitution rate priors.

Maximum Likelihood (ML) analyses were conducted in RaxML v.7.2.8. [65]. Independent GRT+G+I substitution models were assigned to each partition of the ten-partition scheme (see above), and a binary model was applied to the gaps. The best maximum likelihood tree was selected from 100 iterations and support assessed with 1000 replicates of bootstrap resampling.

All trees were visualized and manipulated with the program FigTree v. 1.3.1 [66].

### Estimation of divergence times

Divergence time was estimated using two complementary approaches: concatenation and a coalescent-based approach. Time estimates were conducted in a Bayesian framework with the help of the program BEAST 1.7.4. [67]. The concatenation analysis only included mitochondrial data (*Cox*, *16S, L1* and *nad1*) to facilitate the comparison of *T. canariensis* divergence times with other Canarian ground dwelling spiders reported in the literature. Additionally, distant outgroups were removed from the analyses to reduce branch length disparity (i.e. only *Titanidiops* and *Idiops syriacus* samples were included). In an attempt to facilitate convergence, a simplified partition scheme by gene was implemented with corresponding substitution model provided from PARTIONFINDER. All genes were set to share the same tree and a Yule speciation model was set as the tree prior. Preliminary model comparison using Bayes Factor based on Path Sampling/Stepping stone sampling methods [68] indicated that the alternative Birth-Death model did not provide a better fit to the data.

Because of the lack of fossil data and relevant biogeographic events, we relied on a spider specific mitochondrial substitution rate available in the literature [69]. A normal prior was assigned to the ucld.mean parameter of the lognormal relaxed clock, with initial and mean value 0.0127 and standard deviation 0.0045. Three independent chains of 5×10^7^ generations were run and subsequently analysed using the same procedure as described above (see *Delimitation of putative independent evolutionary lineages* section).

To ascertain the timeframe of the evolution of *Titanidiops canariensis*, a multi-gene coalescent approach as implemented in the program *BEAST [70] was performed. All GMYC lineages of *T. canariensis* with at least two sequences per gene were included. The GMYC 1 clade from *Titanidiops* sp. from Morocco was used as outgroup. Similarly to the concatenated analysis, the partition scheme was simplified to speed up computation and facilitate convergence. Mitochondrial genes were combined in a single partition and a single evolutionary model, lognormal relaxed clock and tree were defined for this partition. Conversely, independent models, lognormal relaxed clocks and trees were allowed for each nuclear gene. Along with mitochondrial substitution rate implemented in the concatenated analysis, a substitution rate for the *EF1γ* was set to 0.00117/per site/million years (normal distribution prior ucld.mean = 0.00117, sdev = 0.00014) as recently estimated for mygalomorph spiders [71]. Uniform priors to the ucld.mean were assigned for the *28S*, *H3* and *AL1*-*Hsp70,* with lower and upper bounds 0.0001 and 0.0115, respectively, and starting value 0.001, under the assumption that the nuclear genes are about one order of magnitude slower than mitochondrial and generally no nuclear protein coding gene will show higher rates than the mitochondrial genes [71], [72]. Finally, we used the oldest subaerial age of Fuerteventura (22 My) [73] as a maximum bound for the tree root. Four independent runs of 100 millions of generations were run remotely on the CIPRES portal [64]. Results were monitored and analysed as already specified in the concatenated analysis.

### Phylogeographic analysis

To infer the phylogeographic history of Canarian *Titanidiops*, we used a Bayesian discrete phylogeographic approach [74] as implemented in BEAST. The Bayesian Stochastic Search Variable Selection (BSSVS) was used to identify the rates, i.e. colonisation pathways, that were frequently invoked to explain the diffusion process [74]. We defined 5 discrete biogeographic areas corresponding to the main independent volcanic edifices that conform the present day emerged parts of the Eastern Islands, namely (from south to north) the Southern (SVC), the Central (CVC) and the Northern Volcanic Complexes (NVC), in Fuerteventura, and Los Ajaches (AJA) and Famara (FAM) in Lanzarote [75] (Fig. 1).

We constructed two new data sets to run the phylogeographic analyses, one with the mtDNA data and a second one with the nuclear *EF1γ*, in both cases including all Canarian specimens. Unfortunately, all the analyses conducted on the nuclear partition reported infinite likelihoods, probably due to the very low divergences among the sequences. Therefore, phylogeographic analyses were restricted to the mtDNA partition. The discrete phylogeographic analyses were run under the uncorrelated lognormal molecular clock for the mtDNA data, specifying the same normal prior for the ucld.mean as above (*Estimation of divergence times*), and a strict clock model for the location trait. The corresponding partition scheme and evolutionary models for the mtDNA were selected with PARTITIONFINDER, and the asymmetric substitution model was assigned to the location. The new matrix included a mix of coalescent (within GMYC clusters) and speciation (between GMYC clusters) level divergences. Therefore we conducted the analyses using the *BEAST option, assigning individuals to their corresponding GMYC cluster but linking all genes to the same clock model and tree. All other settings were identical to those used in the dating analyses.

### BPP

The status of mitochondrial GMYC clusters as candidate species was further tested in a Bayesian multi-species coalescent framework using the species delimitation method in the program BPP 2.2 [76], [77]. This method calculates the posterior probabilities of alternative species delimitation models using a multispecies coalescent approach and interprets gene tree incongruence as the result of ancestral polymorphism.

Following Leaché & Fujita [78], we used the rjMCMC algorithm 0 with the fine-tuning parameter ε set to 15.0. Each species delimitation model was assigned an equal prior probability. Because of the volcanic nature of the Canary Islands, and in the absence of information about the ancestral population size of the target organism, we assumed an evolutionary scenario where the colonising ancestral population was of a small size due to the frequent bottlenecks associated with volcanism and island colonisation (θs *∼G*(2, 2000)). The effects of alternative species divergence time scenarios on species delimitation were tested by implementing either shallow (τ0 *∼G*(2, 2000)) or deep divergence priors (τ0 *∼G*(1, 10)). The species tree obtained in the *BEAST analysis was used as the fixed topology. The species delimitation analyses were restricted to those clades determined with high support (i.e., p>0.95) in the *BEAST analyses (see results). The convergence and sensitivity of the results to the initial condition were assessed by running three independent chains for each parameter combination, each time starting with a different species delimitation model: one that lumped all candidate species into a single species, one that considered an intermediate number of species and one that considered the full range of species.

### Population structure

Standard genetic diversity indices, including the nucleotide (π) and haplotype diversity (H) indices, were calculated in DnaSP 5.10.1 [48] for *cox1*, *EF1γ*, *H3* and *AL1*-*Hsp70* for all GMYC clades consisting of at least two individuals. Additionally, the values were also calculated for the *28S 16S* + *L1* and *nad1* fragments in the dataset, the Moroccan *Titanidiops* and *T. canariensis* samples and the two *T. canariensis* main clades determined in the *BEAST analyses.

Allele networks were constructed in search for the possible geographic patterns in the allele distribution in *EF1γ* and *AL1*-*Hsp70* using the minimum spanning tree method in the program HapStar [79] based on the output provided by Arlequin [80].

We looked for patterns of isolation-by-distance in the Canarian samples using the online version of the program IBDWS 3.23 [81]. The *cox1* F_ST_ pairwise estimates calculated in IBDSW from raw sequences and the geographic distances between the locations obtained in Geographic Matrix Distance Generator v. 1.2.3 (Ersts, American Museum of Natural History, Center for Biodiversity and Conservation, http://biodiversityinformatics.amnh.org/open_source/gdmg) were correlated in three sets of analyses: one including all *T. canariensis* samples and the other two using the two main clades identified in *Beast.

## Results

### Sampling, sequencing and recombination testing

Information about specimens, localities and sequence GenBank accession numbers are listed in S1 Table, and the localities are shown in the map in Fig. 1. A total of 100 specimens were sequenced in the present study. The following gene fragments were obtained for *Titanidiops*: the mitochondrial *cox1* (673 bp, 231 variables), *16S*-*L1* (600 bp, 177 variables) and *nad1* (382 bp, 149 variables) and the nuclear *28S (*762 bp, 22 variables), *EF1γ* (844 bp, 52 variables), *H3* (347 bp, 46 variables) and *AL1*-*Hsp70* (606 bp, 86 variables). No recombination was detected within any of the gene fragments used in this study.

### GMYC-based lineage delimitation

**Figure 3:**
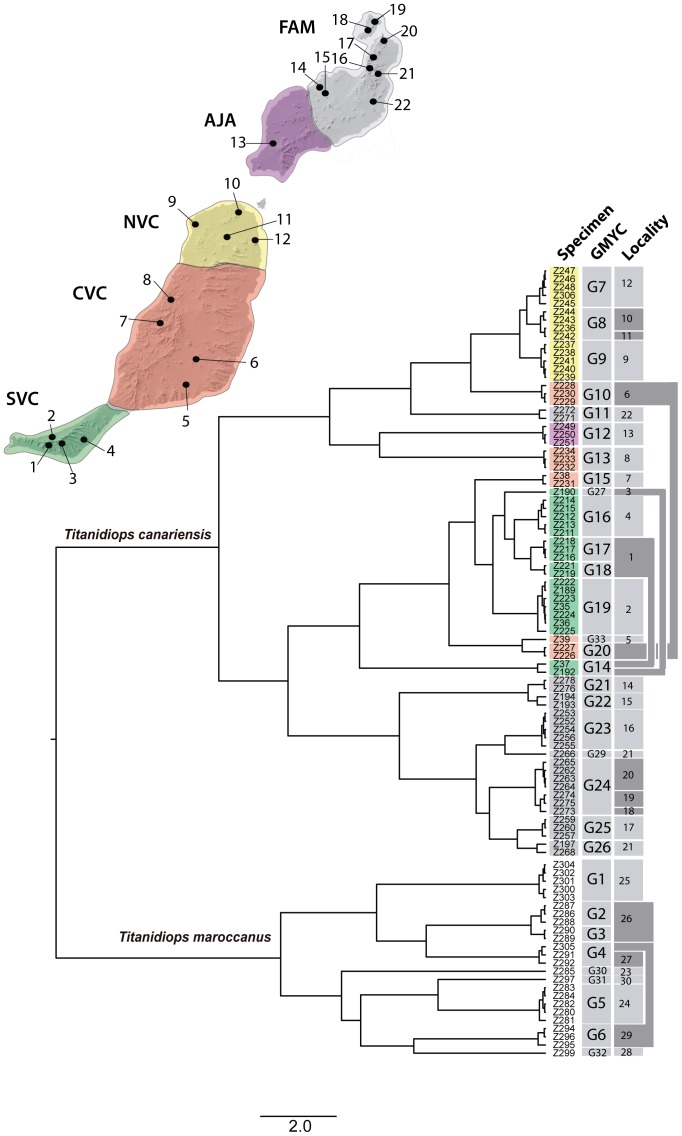
Ultrametric *cox1* BEAST tree. GMYC column: clades identified as independent GMYC clusters in the SPLITS analyses, labelled as in S1 Table. Locality column: localities where the respective GMYC cluster was collected. Localities in dark grey correspond to those either including more than one GMYC cluster or those where the GMYC cluster was found in an additional locality (connected by bars). Terminal colour codes represent geographic location as shown in the map. SF: southern Fuerteventura (Jandia Peninsula), CF: central Fuerteventura, NF: northern Fuerteventura, SWL: south-western Lanzarote, NEL: north-eastern Lanzarote (includes La Graciosa islet).

The complete *cox1* data matrix, including 98 *Titanidiops* specimens from the Canaries and Morocco, was analysed using the single-threshold option of the GMYC algorithm, which was shown not to be significantly worse than the multiple-threshold option (p = 0.23). The GMYC algorithm identified 32 entities/clusters (CI: 27–34) (p = 4.6*10^−9^), of which 9 were Moroccan and 23 were from the Canary Islands (S1 Table, Fig. 3). In most cases, the GMYC clusters corresponded to single localities, and each locality included a single GMYC cluster (62%). Exceptions to this pattern included 4 instances of GMYC clusters found in more than one locality, usually including one or more nearby localities, and 5 instances of localities with more than one GMYC, usually involving closely related clusters. Interestingly, two different GMYC clusters belonging to distant clades (G20 and G10) were sampled from locality 6, in central Fuerteventura.

### Phylogenetic analyses

All genes except *AL1*-*Hsp70*, which could only be reliably amplified and sequenced in the Canarian specimens, were concatenated with the outgroup sequences in a single matrix for subsequent Bayesian and ML tree inference. The concatenated data matrix consisted of 3,583 bp (some alignment positions with a high proportion of missing data were removed) and 32 binary coded gaps scored for 51 terminals. The *Titanidiops canariensis* lineages were represented by single representatives of each GMYC cluster to increase the speed of the analyses. The partition scheme and corresponding evolutionary models are summarised in Table 1.

**Figure 4:**
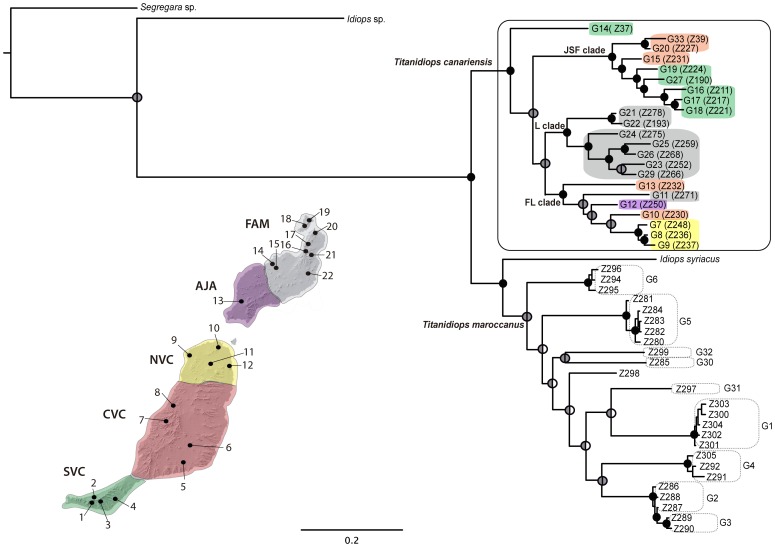
Topology obtained in the concatenated Bayesian analyses. *Titanidiops canariensis* is represented by single representatives from each GMYC cluster identified (Fig. 3, S1 Table). *T. maroccanus* GMYC clusters are highlighted in boxes. Dots on nodes denote support as follows: left semi-circles are Bayesian posterior probabilities (PP) and right ones are maximum likelihood bootstraps, black = PP>0.95, ML bootstrap support >80%, grey  =  clade determined but with support values less than the thresholds above, white  =  topology not determined. Terminal colour codes as in Fig. 3.

The Bayesian and maximum likelihood (-lnL 15343.170107) analyses of the concatenated data matrix resulted in similar tree topologies, although the Bayesian inference yielded higher clade supports (Fig. 4). Both analyses supported the reciprocal monophyly of *T. canariensis* and a clade comprising *Idiops syriacus* and the Moroccan *Titanidiops* lineages. The internal topology of *T. canariensis* was well supported and highly congruent between the methods. Conversely, most relationships within the deeply divergent Moroccan *Titanidiops* lineages were poorly supported.

Both analyses indicated a well-supported clade (hereafter referred as the JSF clade) that includes the specimens from the Jandia Peninsula in southern Fuerteventura except for the individual Z37, which constituted an independent lineage, along with some specimens from central Fuerteventura. These Fuerteventura lineages were shown to be sister lineages to the remaining Canarian representatives, albeit with low support. Similarly, most Lanzarote specimens formed one well-supported clade (hereafter referred as the L clade), including two reciprocal geographic clades (northeast and southwest), which were sisters, albeit with low support, to a clade (hereafter referred as the FL clade) that includes representatives from northern and central Fuerteventura and two additional Lanzarote lineages, one from the southwest and one from the northeast. Although the internal relationships of the last clade were poorly supported, the central Fuerteventura lineage G13 (Z232) was determined to be a sister group to all remaining lineages within the clade.

### Estimation of divergence times

**Figure 5:**
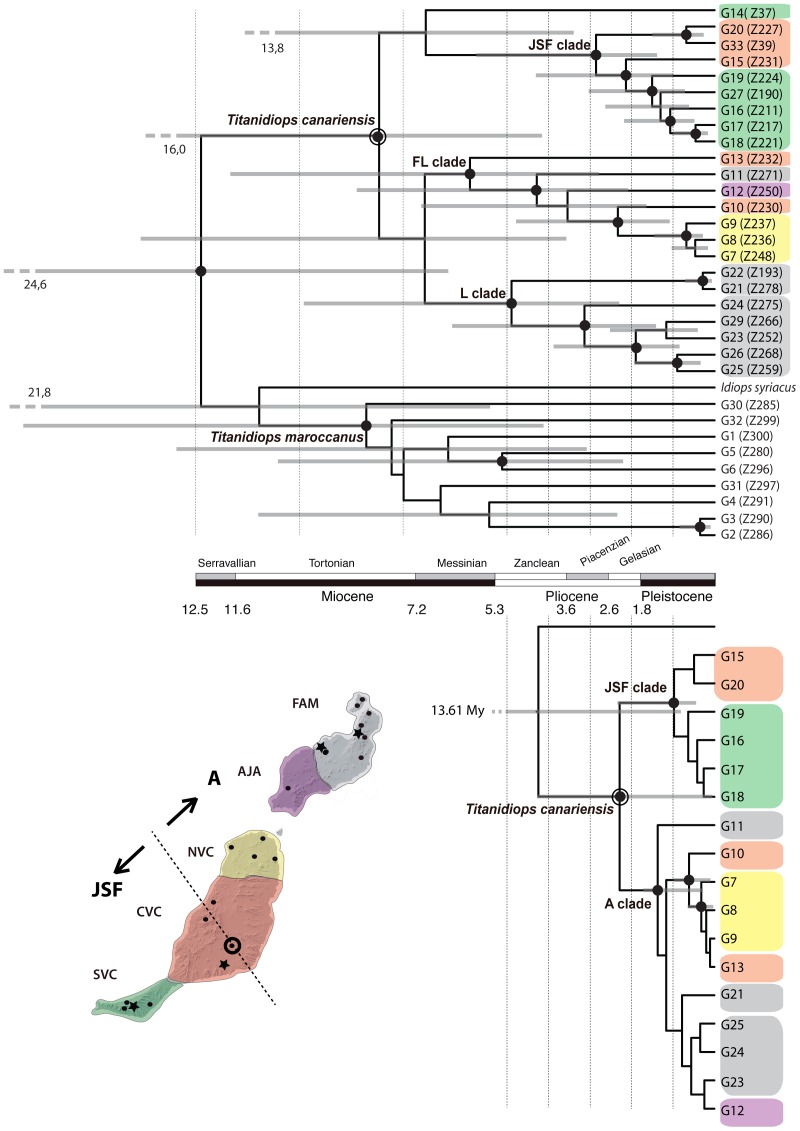
Chronograms obtained with (a) the concatenated approach using BEAST and with (b) the multispecies coalescent (species tree) approach using *BEAST. Dots on nodes denote Bayesian posterior probabilities above 0.95. Node bars indicate the 95% HPD confidence intervals of the divergence time. The common x-axis is time in million years (My). Terminal colour codes are as in the figure inset. Samples from the localities marked as stars on the map were only included in the concatenated approach.

Overall, the tree topology and the clade supports were similar to those found in the Bayesian and ML analyses. The root was assigned to the split between *T. canariensis* and the clade formed by the Moroccan *Titanidiops* lineages and *I. syriacus* (Fig. 5), and it was estimated to have occurred approximately 12 million years ago (Ma) (12.37, 24.65–6.41 Ma). The most recent common ancestor (TMRCA) of *T. canariensis* was estimated at 8.08 Ma (16.01–4.16 Ma). The diversification of the JSF clade began 2.86 Ma (5.74–1.39 Ma), while the L clade and the FL began diversifying earlier, 6.98 Ma (13.82–3.57 Ma).

The split between *Titanidiops maroccanus* and *I. syriacus* was traced back to approximately 11 Ma (10.97, 21.79–5.41 Ma), although this relationship was not well supported. Diversification of the deeper *T. maroccanus* lineages occurred from the late Miocene to the early Pliocene. As in the previous analyses, most relationships within the Moroccan *Titanidiops* remained unresolved.

Special caution should be exercised when interpreting time estimates since they were based on substitution rates obtained from studies analysing interspecific level divergences. It has been shown that the extrapolation of rates across the population-species boundary may overestimate molecular time-scales [82], [83] (but see [84]). In this particular case, however, the pervasive effects of interspecific substitution rates may have been attenuated by the use of single representatives for each mtDNA coalescent clusters (i.e. GMYC clusters).

### Coalescent approach

The coalescent approach resulted in a similar topology to the concatenated analyses but with lower support. The resulting *T. canariensis* species tree (Fig. 5) was divided into two well-supported clades, one corresponding to the JSF clade determined in the previous analyses, and the other one (hereafter referred to as the A clade) included the remaining individuals, which were also determined in the previous analysis albeit with lower support. The internal relationships of the JSF clade corresponded approximately to those found in the previous analyses. Conversely, clade A showed major lineage rearrangements compared to the previous analyses, mostly involving lineages G11, G12 and G13. Lineage G11, which was shown to be part of the clade FL in the previous analyses, was determined to be a sister lineage to the remaining lineages in clade A. The lineage G12, also shown to be part of the FL clade in the previous analyses, was nested within the former L clade. Finally, lineage G13, which was formerly supported as the first split within the FL clade, was nested with high support within a clade formed by the lineages from northern Fuerteventura. Overall, these differences resulted in more geographically congruent clades.

Lineage divergence times were much younger than those determined in the mtDNA concatenated analysis. The origin of the *T. canariensis* stem was placed at 4.25 Ma (13.6–0.8 Ma), while its TMRCA dated back to 2.29 Ma (4.28–0.05 Ma). The diversification of clade A began approximately 1.36 Ma (1.71–0.27 Ma). The origins and diversification of the main geographic lineages, namely the JSF clade, the central-northern Fuerteventura clade and the Lanzarote clade, occurred approximately 1 Ma (2.34–0.44 Ma)

### Phylogeographic analyses

**Figure 6:**
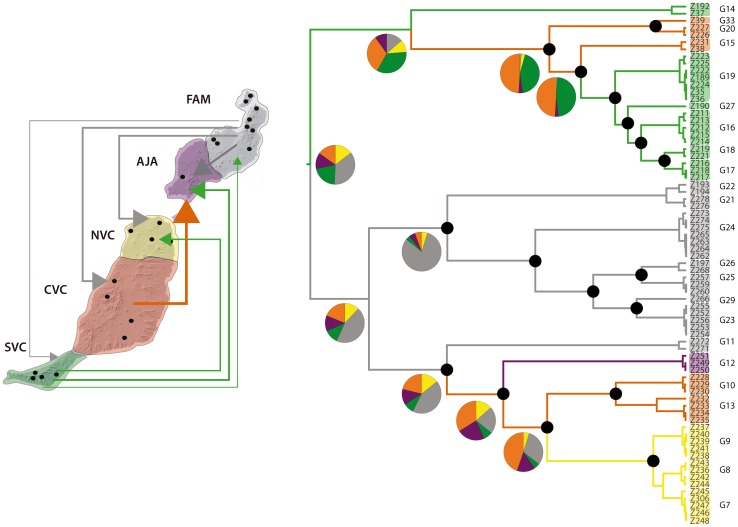
Bayesian ancestral range reconstruction and colonisation history of Canarian *Titanidiops* based on mtDNA markers. Black dotes identify nodes with Bayesian posterior probabilities (PP) >0.95 (all nodes >0.5). Branches coloured as the ancestral range with highest marginal probability for each lineage as inferred in BEAST. Node pie charts represent marginal probabilities for alternative ancestral ranges. Colonisation routes identified by BSSVS are shown on the map with line width proportional to the rate value.

Results of the discrete phylogeographic analyses are summarized in Fig. 6. The extant *T. canariensis* diversity most likely originated in northern Lanzarote (FAM). However, this result is to be considered as tentative due to the low support for the basal node and the low posterior probability of the area assignment. A subsequent split separated central-southern Fuerteventura (SVC, CVC) and northern Lanzarote (FAM) lineages. Southern Lanzarote (AJA) and northern Fuerteventura (NVC) were colonised form northern Lanzarote (FAM). Central Fuerteventura (CVC) was probably colonised at least two times independently, from northern Lanzarote and from Southern Fuerteventura (SVC).

### BPP

The following lineages, delineated with high support in the *BEAST analysis, were included in the species delimitation analyses: G15+G20 (candidate species 1, sp. 1), G16-G19 (sp. 2), G11 (sp. 3), G10, (sp. 4) and G7-G9+G13 (sp. 5). In addition, although not supported, the clade G21+G23-G25 (sp. 6) was also tested, as it was the sister group of a well-supported clade and similar in age to the remaining clades analysed.

**Figure 7:**
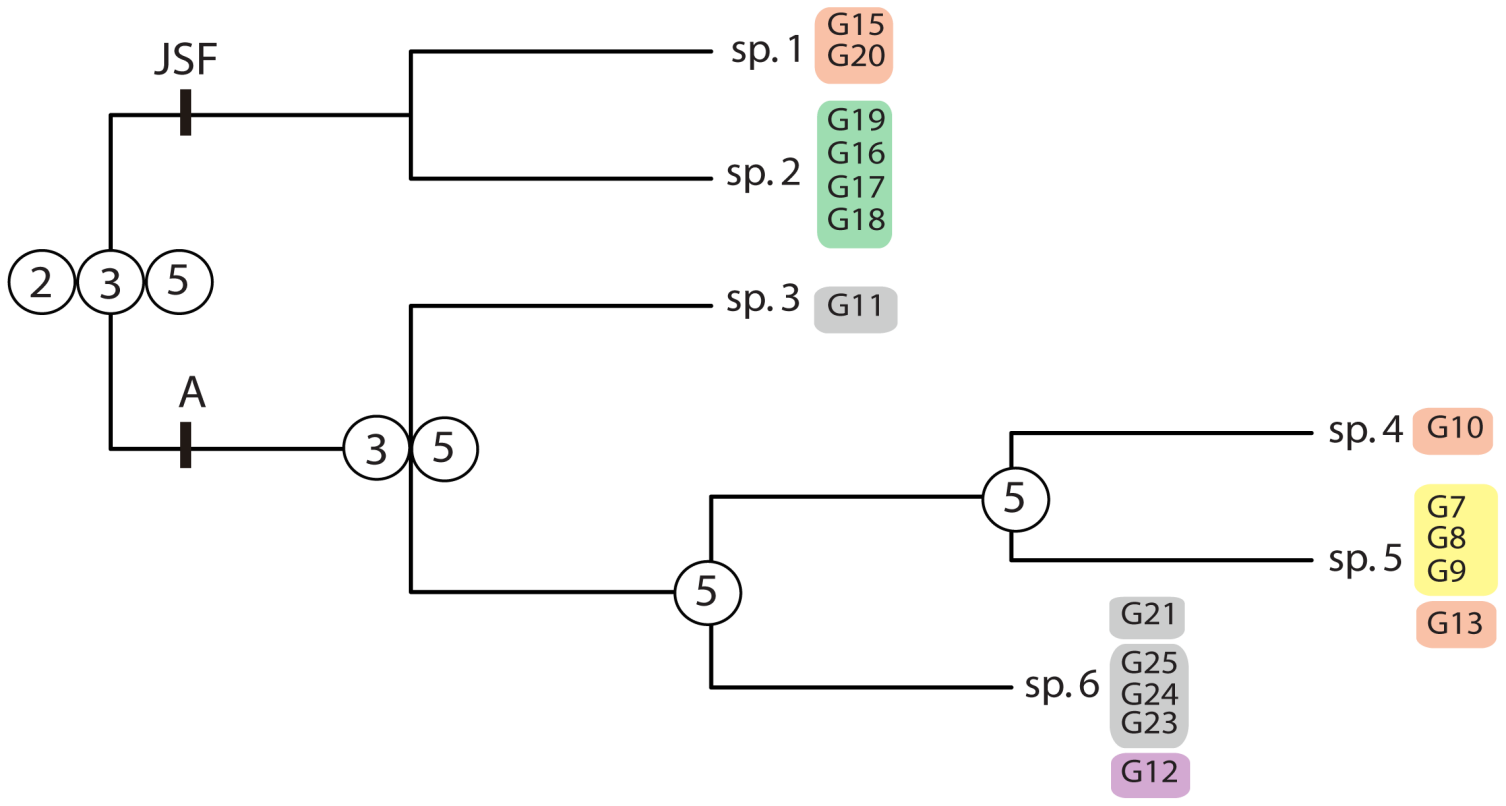
Multi-locus Bayesian species delimitation with BPP. Only results obtained under the small population sizes (θs *∼G*(2, 2000)) and shallow divergence (τ0 *∼G*(2, 2000)) scenarios are shown. Circles on nodes indicate lineages (descendants) supported as independent evolutionary lineages (i.e., species) and the total number of independent lineages supported under alternative models of number of starting species (K). Terminal colour codes as in Fig. 3.

Under the first scenario tested, i.e., shallow divergences, all runs starting from K = 1 and K = 2 supported (i.e., a posterior probability of the split >0.95) a two species hypothesis, in agreement with the basal split between the JSF and A clades (sp1+sp2 and sp3+sp4+sp5+sp6). Runs starting from K = 3 resulted in three delimited species because clade A was further divided into sp3 and sp4+sp5+sp6. Finally, runs under K = 4, K = 5 and K = 6 supported a five species hypothesis, sp1+sp2, sp3, sp4, sp5 and sp6 (Fig. 7). In all cases, sp1 and sp2 were lumped into a single species. The results of the second scenario, deep divergences, were directly dependent on the selected K values. These results were identical to the defined starting models.

### Population analyses

Standard genetic diversity indices, nucleotide diversity (π) and haplotype diversity (H), were calculated for the *cox1*, *EF1γ*, *H3* and *AL1*-*Hsp70* genes for each GMYC lineage comprising two or more individuals from the entire *Titanidiops* dataset, for *T. canariensis*, *T. moroccanus* and for the JSF and A clades. The results are summarised in S2 Table.

The *T. canariensis EF1γ* allele network (S1 Figure) included 27 unique alleles. The two most common alleles, which differed by a single substitution and were shared by 18 and 12 individuals, were collected from all geographical zones. Overall, the network was congruent with the main mtDNA lineages, assuming the most frequent alleles were shared across lineages due to ancestral polymorphism.

The resulting *AL1*-*Hsp70* network (S2 Figure) contained 45 distinct alleles. The highest allele diversity was found among the northern Lanzarote samples (20 alleles), and the northern Fuerteventura samples showed the lowest (3 alleles). The remaining three geographic zones presented a moderate number of alleles (central and southern Fuerteventura had 10 alleles, respectively, and south-western Lanzarote had 7). Unlike the *EF1γ* network, the *AL1*-*Hsp70* one had little geographic structure, and most mtDNA lineages were not determined. The JSF clade was an exception, and the alleles belonging to individuals carrying this mtDNA lineage were all closely related. A group of alleles found mostly in northern Lanzarote (one from southern Lanzarote) was separated from all remaining alleles by at least 14 missing mutations.

### Isolation by distance

There was no significant correlation between genetic and geographic distances, either for all *T. canariensis* samples together or for any of the two main lineages (JSF and A) (r (P) = −0.0036 (0. 5746), 0.2879 (0.3391), −0.0710 (0.7247), respectively).

## Discussion

### Mygalomorph spiders on the oceanic islands

Mygalomorph spiders are notoriously absent from oceanic archipelagos, most likely due to their low dispersal abilities [23], [24], [26], [85]. The very few reported exceptions include members of the ctenizid genus *Ummidia* in the Caribbean, one of the few mygalomorphs that uses airborne dispersion. Several species of the family Barychelidae, presumably without ballooning capability, are found on remote oceanic archipelagos in the Pacific [86], [87], [88], but no explicit biogeographic hypotheses have been put forward to explain their origin. Here, we provide, for the first time, dated phylogenetic information to decipher the origins of an oceanic mygalomorph.

In spite of the relatively short distance between the Canary Islands and northern Africa, approximately 110 km at the narrowest point, the well-supported monophyly of the Canarian lineages suggests a single event of colonisation of the Canary Island by *Titanidiops*. Although the mechanism by which *Titanidiops* colonised the Canaries remains speculative, it has been suggested that the ground-dwelling spider *Dysdera* colonised the Canary Islands from the neighbouring north-eastern African coast by transporting itself on floating islands [2]. Some of the *Titanidiops* localities sampled in Morocco are distributed around the valley of the Sous River and it is possible that in the more humid climate of the past [89], [90], the river had torrential currents, facilitating the formation of large mats that may have acted as rafts for ground-dwelling arthropods. In addition, sea level regressions [91] would have further facilitated dispersal by reducing the distance from the continent, as suggested by the arrival of the extinct endemic rodent *Malpaisomys* [92].

A large gap was observed between the timeframes of *Titanidiops* diversification determined from only the concatenated mitochondrial data and from the multi-locus species tree approaches. In general, longer divergence time estimates are expected from gene trees because a fraction of gene divergence may pre-date population/species divergences [93], [94]. There is ample empirical evidence that the use of species-tree approaches usually results in younger estimates of species divergence times [95], [96], [97], sometimes as much as three times younger [98]. In our study, the species tree estimates of the timing of most recent common ancestor of the Canarian *Titanidiops* (2.29 Ma, 4.28–0.05 Ma) were about half as young as the mtDNA gene estimates (8.08 Ma, 16.01–4.16 Ma). Differences in time estimates under both approaches may be exacerbated in the case of large ancestral populations [96] or in the presence of gene flow among lineages [95]. Because of the inherently small populations involved in colonisation events, in the case of *Titanidiops*, the existence of gene flow may provide a better explanation. The effect of such events may also explain some of the incongruences observed in the *AL1*-*Hsp70* allele network (S2 Figure).

The mismatch between the estimated times using the two approaches, either during the Miocene or in the Pliocene, hinders the proposal of a geological scenario for the colonisation of the islands. Although the random nature of the colonisation process could explain the absence of *Titanidiops* from the western Canaries, the strong environmental differences between the arid and low-lying Eastern Canaries and the lush and elevated Western Canaries may suggest specific ecological or habitat preferences. If *Titanidiops* are better suited to the present day environmental conditions in the Eastern Canaries, its arrival to the island probably occurred after the onset of such conditions.

It is worth noting that there are other cases of eastern Canarian endemic lineages with northern African close relatives, especially among plants [99]. Examples of faunal connections include the spider *Dysdera lancerotensis* [100], the *Calathus* beetles [12], the *Chalcides* skinks [101], the *Tarentola* geckos [11] and the fossil rodent *Malpaisomys* [92]. Available time estimates for these lineages suggest a mid-Pliocene to early Pleistocene colonisation of the Eastern Canaries. Interestingly, this timeframe coincides with the onset of a major dryness event in the region following the establishment of the cold Canarian sea current ∼4 Ma [102]. Our species-tree estimates of the Canarian *Titanidiops* stem fits well with the former scenario, which would suggest a pre-adaptation to xeric conditions. However, the time estimates in the cited studies were obtained either from single mitochondrial genes or from gene concatenation.

Our concatenated *Titanidiops* divergence time estimates are much older, tracing back to the Miocene epoch, and are closer to the values reported for the Canarian lineages that have undergone species radiations, such as the *Pholcus* [103] and most of the *Dysdera* [4], [104] spiders and the *Gallotia* lizards [105]. Similarly, the coalescent time of *Titanidiops* (4.25 Ma, 13.60–0.80 Ma) pre-dates most values reported in the literature for Eastern Canaries endemics, including the lizard *Gallotia atlantica* (1.9 Ma, confidence interval 3.3–1.3 Ma) [7], the darkling beetle *Hegeter politus* and the spiders *Dysdera lancerotensis* (1.5 Ma, 2.3–0.7 Ma) [100], *D. nesiotes* (0.8 Ma, 1.1–0.53 Ma) and *D. alegranzaensis* (0.83–0.4 Ma) [5].

Further support for the old (Miocene) colonisation of the Canaries by *Titanidiops* may come from the observation that the sampled population from the continental source, Morocco, formed a monophyletic group, indicating that the continental lineages that gave rise to the island populations may have gone extinct and, hence, that the colonisation preceded the current diversification of *T. maroccanus*. If *T. canariensis* had colonised the Canaries in much more recent times, as suggested by the multi-coalescent analyses, there would have been higher chances to find some descendants of the ancestral continental stock, which would have rendered the continental lineages paraphyletic regarding the island ones.

### How many species of *Titanidiops* inhabit the Canary Islands?

Mygalomorph spiders are phenotypically conservative and pose a challenge to classical taxonomy. The application of molecular tools to investigate phylogeographic patterns in this group has uncovered a great amount of hidden diversity [23], [36], [85], [106], [107], [108]. However, because of their low dispersal capability and burrow fidelity, mygalomorph spiders tend to form clustered aggregations [109], [110], which usually lead to extensive genetic structures [24], [27], [106], [111], making it difficult to distinguish between fragmented populations and independent evolutionary lineages [26].

Although it is generally agreed that species delimitation should be based on the integration of multiple lines of evidence (i.e., genotypes, phenotypes, ecology) [112], novel statistical approaches for the quantitative assessment of species boundaries mostly focus on genetic data [113], [114]. Among the multiple methods developed to determine species boundaries based on multi-locus data, the Bayesian multi-species coalescent model in the program BPP has become increasingly popular [26], [78], [115], [116], [117], [118], [119]. Although the method assumes no gene flow, efficient performance has been proven with the low gene flow level of 0.1 migrants per generation [120].

In this study, we have applied the BPP method to validate whether the large number of genetically divergent lineages revealed by the GMYC approach corresponds to distinct evolutionary lineages. We tested two alternative scenarios, corresponding to either shallow or deep divergences between candidate species. Because of the dynamic nature of volcanic landscapes, involving recurrent extirpations and recolonisations of populations, and the low vagility of mygalomorph spiders, small ancestral population sizes were assumed for both scenarios. However, given the inconsistent results from the deep divergences scenario and the relatively recent estimates from the species-tree approach for the diversification of *T. canariensis*, which were mostly confined to the Pleistocene, we argue that the scenario assuming shallow divergences between the candidate species is probably the most appropriate.

Our results are sensitive to the starting species number model (K), which, to the best of our knowledge, has not been reported in any previous study. The basal split of *T. canariensis* into two species was the only consistently supported alternative across all starting species models. Higher numbers of species were only supported by particular K values. The BPP method has been found to over split species diversity, most likely because it does not integrate over the species tree parameter space [26]. Here, we take a conservative approach and propose that *T. canariensis* includes two distinct species.

Interestingly, although the two putative species are, for the most part, geographically isolated, they do overlap in at least one locality, suggesting similar habitat preferences. Although the results do not show clear evidence of gene flow, most shared alleles may be explained by ancestral polymorphism, a larger sample and more variable nuclear markers would be required to assess the actual level of genetic isolation.

The “JSF” species was found in all localities sampled on the Jandia peninsula. This massif is effectively isolated from the rest of Fuerteventura by a low-elevation isthmus, covered by aeolian sands, which explains the presence of several examples of vicariant species on the peninsula [121]. However, it was also found in localities across the isthmus that do not show any obvious geographic discontinuities with the populations of the second species, including the co-occurring locality. The branching pattern determined in the better-sampled concatenated analyses is compatible with at least two rounds of back-and-forth colonisation between the main island and Jandia. Therefore, the north-wise displacement of the contact zone between the two putative species could be explained by a secondary colonisation of the Fuerteventura main island from a lineage originally isolated in the peninsula.

It is not strange that all the specimens collected in the present study were either females or juveniles, because they were mostly extracted from burrows, which adult males are known to abandon after the final moult. In fact, only 3 male specimens of *T. canariensis* collected in pitfall trap in Jandia are known at present. Preliminary observations revealed subtle differences in morphology between the putative species; however, in the absence of male material from localities throughout the Eastern Canaries, we have refrained from formally describing a new species.

### Phylogeographic patterns in *T. canariensis*

As was expected based on former phylogeographic studies on trap-door spiders [26], [27], [111], a high number (23) of narrowly distributed mtDNA GMYC clusters, found frequently in single localities, were determined in *T. canariensis*. The GMYC clusters were often formed by individuals sharing identical or very closely related haplotypes (see S2 Table for details). This observation corroborates the tendency of *T. canariensis* to form aggregates, which has also been reported in other trap-door spiders such as the genus *Cyrtocarenum* from the family Ctenizidae [109]. These results provide further support for the limited dispersal ability of trap-door spiders. Although low vagility usually promotes isolation by distance, which has been suggested to play an important role in speciation in mygalomorph spiders [23], [36], [85], [106], [107], [108], there was no evidence of such a pattern in *T. canariensis*. The explanation for this lack of correlation may be found in the dramatic relief of the islands. Close localities are frequently separated by steep ridges or deep valleys, which most likely represent an effective barrier to dispersal.

In spite of its low dispersal ability, *T. canariensis* successfully spread through the Eastern Canary Islands. The Bayesian phylogeographic analysis identifies northern Lanzarote (FAM) as the ancestral range for *T. canariensis* and suggests at least two independent colonisation of Fuerteventura and probably a back colonisation of southern Lanzarote from Fuerteventura. Similar complex phylogeographic patterns within Lanzarote and between Lanzarote and Fuerteventura have been reported in other Eastern Canaries endemic taxa [5], [7], [100] and have been explained as the consequence of frequent population extinctions due to lava flows and the recurrent connections of the two islands during Neogene marine transgressions. It is noteworthy that one of the Lanzarote lineages includes a locality in the Zonzamas area (G11, locality 22), which has been identified as a refugium during episodes of volcanic activity [3], [5], [7].

Our results also suggest a recent colonisation of La Graciosa from Lanzarote, as samples from La Graciosa belong to the same GMYC cluster (G24) as those from the nearby locality of Mirador del Rio in Lanzarote. La Graciosa is separated from Lanzarote by a narrow seaway approximately 2 km wide and of shallow water, and most of the shelf connecting the islands was exposed during the Pleistocene sea level oscillations [18], facilitating terrestrial dispersal between the two islands. This pattern differs from that observed in *D. lancerotensis*, for which La Graciosa populations seem to have originated from the northern islet of Alegranza [100]. The La Graciosa populations of *D. alegranzaensis,* conversely, show genetic affinities with both their Alegranza and Lanzarote populations [5].

### Insights into continental *Titanidiop*s diversity

Although determining the diversity and phylogenetic relationships of the continental *Titanidiops* were beyond the scope of the present study, our results provide some insights into the evolutionary patterns of *T. maroccanus.* Several highly divergent lineages were identified within *T. maroccanus*, showing a similar diversification timeframe to that estimated for *T. canariensis*, which may suggest the existence of cryptic species in Morocco. The divergence times estimated within *T. maroccanus* are far older than those reported in any other organism inhabiting the region. The basal split into the two main lineages of the wolf spider species complex *Lycosa oculata*, which comprises five putative species, occurred approximately 3.26 Ma, while present day species diversified between 2.96 to 1.51 Ma [122]. Similarly, most genetic diversity within Moroccan reptile species has been dated to the period of Pleistocene climatic oscillations (starting at 2.3 Ma) [123], [124].

The concatenated analyses supported the sister group relationship of the Moroccan *Titanidiops* lineages with *Idiops syriacus* (O.P. Cambridge 1870). Interestingly, *I. syriacus,* although originally described as *Idiops* (Perty 1833), was transferred to the genus *Titanidiops* by E. Simon [125], where it remained until Raven [22] synonymised both genera. When the genus *Titanidiops* was re-erected by J. Wunderlich [34], [35], the placement of *I. syriacus* and other former members of *Titanidiops* was not indicated. Interestingly, in the original description of *T. maroccanus*, Simon had already indicated that *I. syriacus* was the most similar species to the Moroccan *Titanidiops* [126]. Our results confirm this observation. However, additional representatives of the genus *Idiops*, and other genera within the family Idiopidae will be required to establish the phylogenetic limits of the genus *Titanidiops* and generic placement of *I. syriacus*.

Interestingly, the distribution of *Titanidiops* closely matches the continental floristic disjunction known as the “Rand” pattern [127], [128]. Many plant lineages show disjunct distributions between Macaronesia/northwest Africa, the Horn of Africa/southern Arabia and east/south Africa. A vicariant hypothesis was put forward to explain this pattern, which would be the result of the partial extinction of an ancient widespread African flora driven by post-Miocene aridification [129]. Recent analyses, however, suggest that dispersal may have also played an important role in shaping diversity in some of the areas involved [128]. The inclusion of the East-African *Titanidiops* species in future analyses will enable to test alternative hypothesis on the origin of the “Rand” biogeographic pattern in invertebrates.

The sister group relationship of *T. maroccanus* and *I. syriacus* has important implications for inferring the origins of the Canarian *Titanidiops*. The origin of *T. maroccanus* postdates the split of *T. canariensis* from their common ancestor and, most likely, the colonisation of the Canary Islands. This observation suggests that present day Moroccan diversity may be the result of a secondary colonisation of the region by the *Titanidiops* lineage. Alternatively, the Canaries could have acted as the source area for the subsequent colonisation of the continent, following a west to east progression pattern. Back colonisation of the continent from the Macaronesian islands has been documented in several groups, including plants, birds and invertebrates [130], [131], [132].

## Conclusions

*T. canariensis* colonised the Canary Islands once, presumably from northern Africa, although no closely related Moroccan *Titanidiops* lineage has been detected. The time of colonisation remains undetermined, mostly due to the discrepancy between the time estimates obtained with the concatenated and the species tree approaches. Several lines of evidence, however, point towards a Miocene origin. A complex phylogeographic pattern was revealed, involving two independent colonisations of Fuerteventura from the ancestral range on northern Lanzarote and probably a back colonisation of southern Lanzarote from Fuerteventura. Volcanic activity may have contributed to the determined geographic patterns of genetic diversity, which further supports the existence of a volcanic refugium in the Zonzamas area in west-central Lanzarote. Our results are compatible with the existence of at least two species in the Canaries, one inhabiting the Jandia Peninsula and southern Fuerteventura and the second ranging from central Fuerteventura to northern Lanzarote. The two species co-occur in at least one locality.

Several highly divergent lineages were also detected within *T. maroccanus*, most likely representing cryptic species. In this study, a close phylogenetic affinity between the Moroccan *Titanidiops* lineages and *Idiops syriacus* was also uncovered, in agreement with the previous placement of *I. syriacus* in the genus *Titanidiops*, which suggests either a different origin for the Canarian and the present day Moroccan lineages of *Titanidiops*, or a back colonisation of the continent.

## Supporting Information

S1 Figure **EF1 g allele network.** Circle size is proportional to the allele frequency. Small filled circles represent missing alleles. Each allele is labelled with the individuals and GMYC cluster in which it was found. Asterisks indicate samples from the single locality (6) where the two putative species co-occurred (G10, G20).
(TIF)

S2 Figure ***AL1*** **-** ***Hsp70*** **allele network.** Circle size is proportional to the allele frequency. Small filled circles represent missing alleles. Each allele is labelled with the individuals and GMYC cluster in which it was found. Asterisks indicate samples from the single locality (6) where the two putative species co-occurred (G10, G20).
(TIF)

S1 Table **Specimen information, locality data and GenBank accession numbers.**
(DOC)

S2 Table **Standard genetic diversity indices, nucleotide diversity (π) and haplotype diversity (H) of the** ***cox1*** **,** ***EF1γ*** **,** ***H3*** **and** ***AL1*** **-** ***Hsp70*** **genes for each GMYC lineage formed by two or more individuals for the entire** ***Titanidiops*** **dataset,** ***T. canariensis*** **,** ***T. maroccanus*** **and the JSF and A clades.**
(DOC)
