# Supplementary figures and images for: Spiders on a Hot Volcanic Roof: Colonisation Pathways and Phylogeography of the Canary Islands Endemic Trap-Door Spider Titanidiops canariensis (Araneae, Idiopidae)
Source: PLoS One. 2014 Dec 10;9(12):e115078. doi: 10.1371/journal.pone.0115078 (PMC4262472; doi:10.1371/journal.pone.0115078)

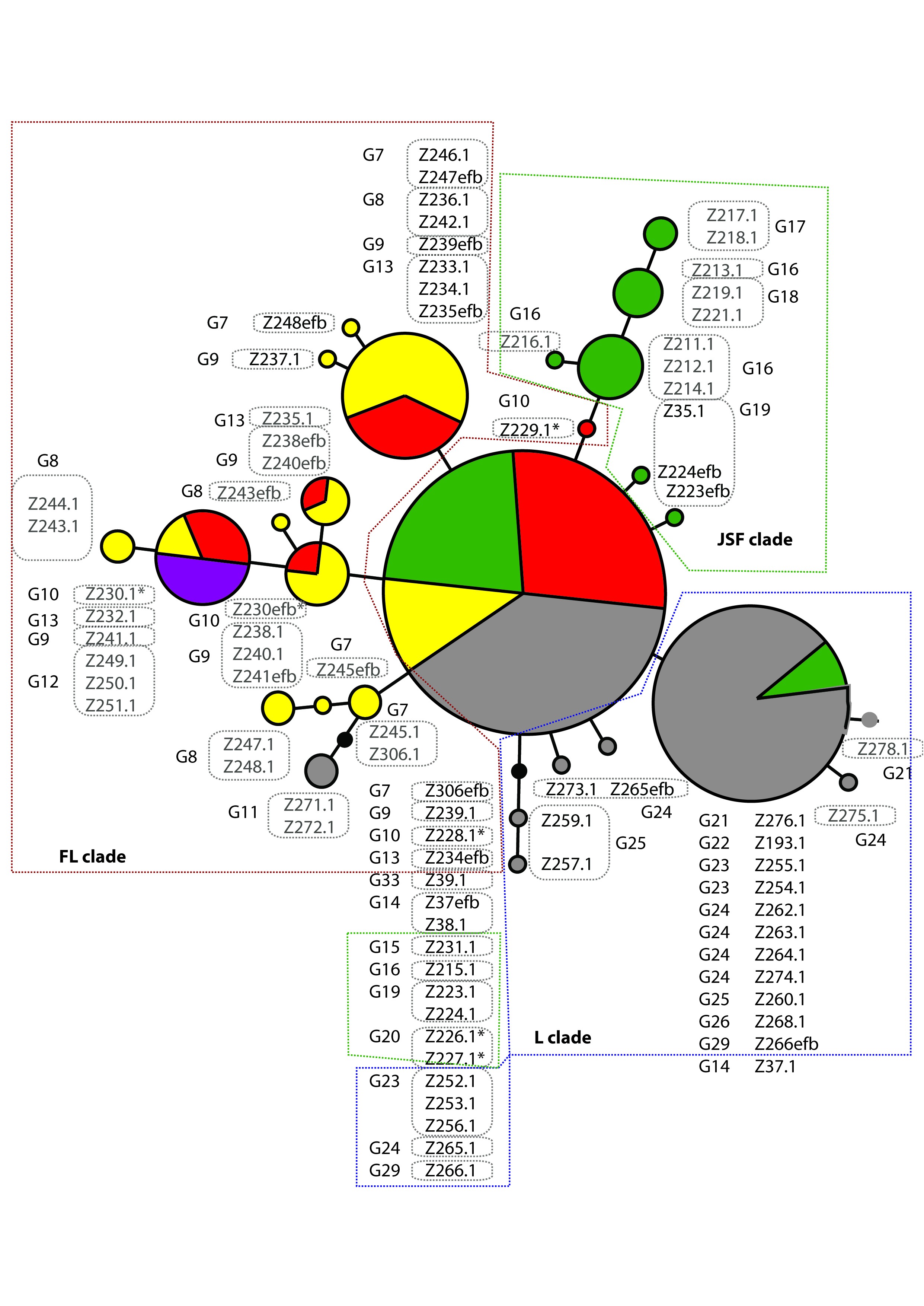

Supplement: S1 Figure — EF1 g allele network. Circle size is proportional to the allele frequency. Small filled circles represent missing alleles. Each allele is labelled with the individuals and GMYC cluster in which it was found. Asterisks indicate samples from the single locality (6) where the two putative species co-occurred (G10, G20). (TIF) [file pone.0115078.s001.tif]

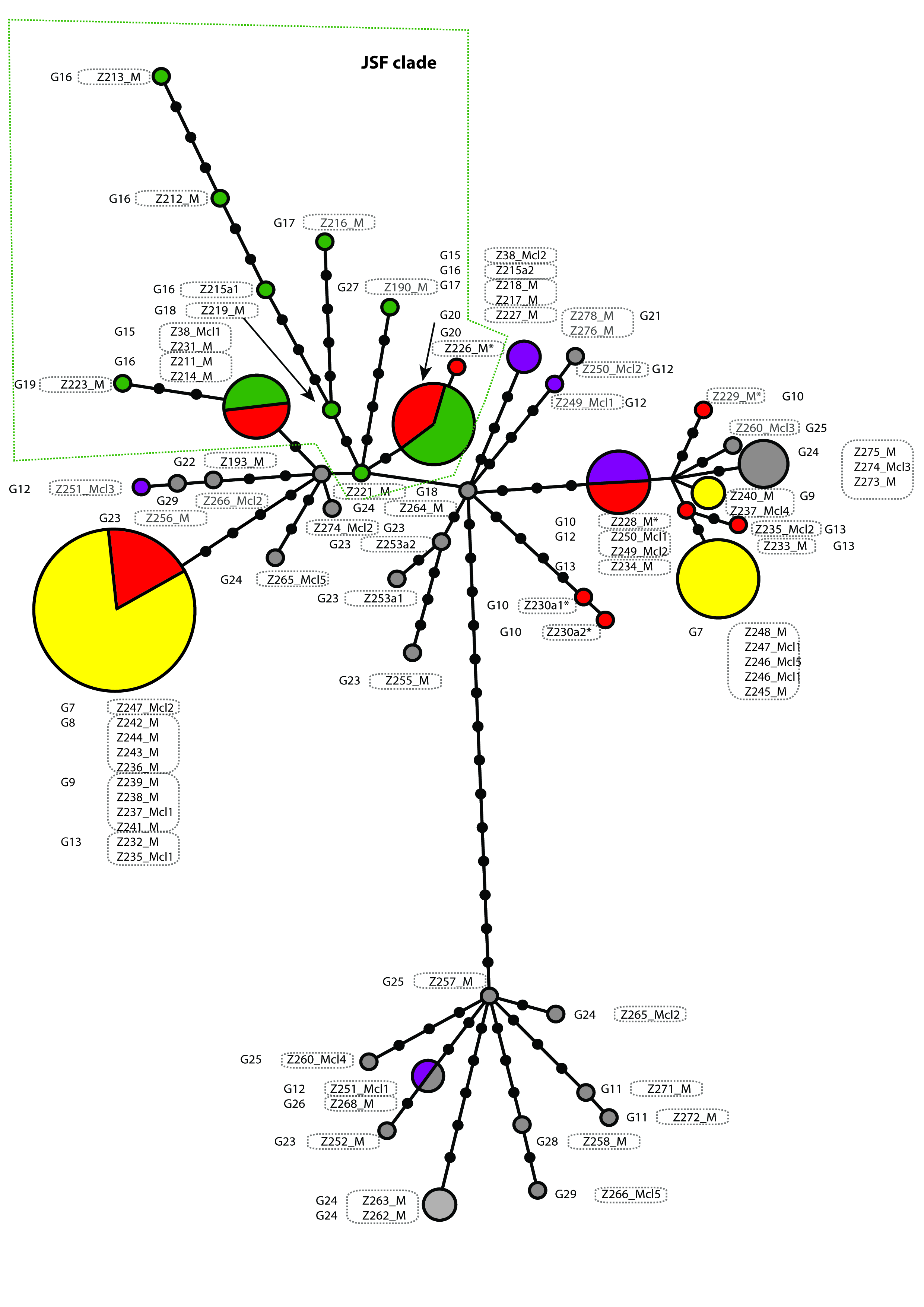

Supplement: S2 Figure — AL1 - Hsp70 allele network. Circle size is proportional to the allele frequency. Small filled circles represent missing alleles. Each allele is labelled with the individuals and GMYC cluster in which it was found. Asterisks indicate samples from the single locality (6) where the two putative species co-occurred (G10, G20). (TIF) [file pone.0115078.s002.tif]
