# Supplementary material for: Spiders on a Hot Volcanic Roof: Colonisation Pathways and Phylogeography of the Canary Islands Endemic Trap-Door Spider Titanidiops canariensis (Araneae, Idiopidae)
Source: PLoS One. 2014 Dec 10;9(12):e115078. doi: 10.1371/journal.pone.0115078 (PMC4262472; doi:10.1371/journal.pone.0115078)
Supplement: S1 Table — Specimen information, locality data and GenBank accession numbers. (DOC) [file pone.0115078.s003.doc]

| Locality name/  Locality number |  | Geographic code | Lat/Long | N | GMYC | Sample code | cox1 | 16s  16s-L1* | nad1 | EF1g | 28S | H3 | AL1-Hsp70 |
| --- | --- | --- | --- | --- | --- | --- | --- | --- | --- | --- | --- | --- | --- |
| Midreshet Ben-Gurion |  | Israel | 30.85179N  34.77654W |  | *Idiops siriacus* | Z579 | KM110419 | KM110687* | KM110709 | KM110522 | KM110319 | KM110595 | --- |
|  |  | South Africa | ­--- |  | *Segregara* | Z912 | KM110420 | --- | --- | KM110523 | KM110320 | KM110596 | --- |
|  |  | South Africa | --- |  | *Idiops* | MY189 | --- | --- | --- | --- | DQ639920 | --- | --- |
| Kemis-oulat-el-Hadj | 24 | Morocco | 31.3964N  9.31604W | 5 | G5 | Z280  Z281  Z282  Z283  Z284 | KM110393  KM110394  KM110395  KM110396  KM110397 | KM110255  KM110256  ---  ---  --- | ---  ---  ---  ---  --- | KM110494  KM110495  KM110496  KM110497  KM110498 | KM110304  KM110305  ---  ---  --- | KM110578  KM110579  ---  ---  --- | ---  ---  ---  ---  --- |
| Smi-Mou | 23 | Morocco | 31.50096N  9.6158W | 1 | G30 | Z285 | KM110398 | KM110257 | --- | KM110499 | KM110306 | KM110580 | --- |
| Jbele Amsittene | 26 | Morocco | 31.15739N  9.69767W | 5 | G2 | Z286  Z287  Z288 | KM110399  KM110400  KM110401 | KM110258  KM110259  KM110260 | ---  ---  --- | KM110500  KM110501  KM110502 | KM110307  KM110308  --- | KM110581  KM110582  --- | ---  ---  --- |
|  |  |  |  |  | G3 | Z289  Z290 | KM110402  KM110403 | KM110261  KM110262 | ---  --- | KM110503  KM110504 | KM110309  KM110310 | KM110583  KM110584 | ---  --- |
| Tamanat - Aid-Beoude rd. | 27 | Morocco | 31.00613N  9.59705W | 2 | G4 | Z291  Z292 | KM110404  KM110405 | KM110688*  --- | KM110710 | KM110505  KM110506 | KM110311  --- | KM110585  --- | ---  --- |
| Imoza rd.  nr. Tourarin | 29 | Morocco | 30.64256N  9.70562W | 1 | G4 | Z305 | KM110417 | KM110268 | --- | KM110519 | KM110318 | KM110592 | --- |
|  |  |  |  | 3 | G6 | Z294  Z295  Z296 | KM110407  KM110408  KM110409 | KM110263  ---  KM110264 | ---  ---  --- | KM110508  KM110509  KM110510 | KM110312  ---  KM110313 | KM110586  ---  KM110587 | ---  ­---  --- |
| Ait-Aisa | 30 | Morocco | 30.17806N  8.29407W | 1 | G31 | Z297 | KM110410 | KM110265 | --- | KM110511 | KM110314 | KM110588 | --- |
| Aid-Baha | 31 | Morocco | 29.84522N  8.93745 | 1 | Not assigned | Z298 | --- | --- | --- | KM110512 | --- | --- | --- |
| Iguer rd. | 28 | Morocco | 30.90627N  8.31932W | 1 | G32 | Z299 | KM110411 | KM110685* | KM110707 | KM110513 | KM110315 | KM110589 | --- |
| Ouzoud Falls rd. | 25 | Morocco | 31.95973N  6.76811W | 5 | G1 | Z300  Z301  Z302  Z303  Z304 | KM110412  KM110413  KM110414  KM110415  KM110416 | KM110266  KM110267  ---  KM110686*  --- | ---  ---  ---  ---  KM110708 | KM110514  KM110515  KM110516  KM110517  KM110518 | KM110316  KM110317  ---  ---  --- | KM110590  KM110591  ---  ---  --- | ---  ---  ---  ---  --- |
| Barranco de Mal Nombre | 4 | SF | 28.09139N  14.28589W | 5 | G16 | Z211  Z212  Z213a  Z213b  Z214  Z215a  Z215b | KM110332  KM110333  KM110334  KM110335  KM110336 | KM110674*  ---  KM110230  ---  --- | KM110696  ---  ---  ---  --- | KM110423  KM110424  KM110425  KM110426  KM110427 | KM110270  ---  KM110271  ---  --- | KM110524  ---  KM110525  KM110526  ---  --- | KM110599  KM110600  KM110601  KM110602  KM110603  KM110604 |
| Barranco del Ciervo | 1 | SF | 28.0854N  14.37196W | 6 | G17 | Z216a  Z216b  Z217  Z218 | KM110337  KM110338  KM110339 | KM110231  KM110675*  --- | ---  KM110697  --- | KM110428  KM110429  KM110430 | KM110272  KM110273  --- | KM110527  KM110528  KM110529  --- | KM110605  KM110606  KM110607 |
|  |  |  |  |  | G18 | Z219  Z221a  Z221b | KM110340  KM110341 | KM110232  KM110676* | ---  KM110698 | KM110431  KM110432 | KM110274  KM110275 | KM110530  KM110531  KM110532 | KM110608  KM110609 |
|  |  |  |  |  | G14 | Z37 | KM110323 | KM110669* | KM110691 | --- | --- | --- | --- |
| Pico de Fraile | 3 | SF | 28.10178N  14.35557W | 2 | G14 | Z192 | KM110328 | KM110228 | --- | --- | --- | --- | --- |
|  |  |  |  |  | G27 | Z190 | KM110327 | KM110672* | KM110694 | --- | --- | --- | --- |
| Cofete | 2 | SF | 28.10265N  14.38232W | 7 | G19 | Z35  Z36  Z189  Z222  Z223a  Z223b  Z224a  Z224b  Z225a  Z225b | KM110321  KM110322  KM110326  KM110342  KM110343  KM110344  KM110345 | KM110667*  KM110668*  ---  ---  KM110233  KM110677*  KM110234 | KM110689  KM110690  ---  ---  ---  KM110699  --- | KM110421  ---  ---  ----  KM110433  KM110434  KM110435  KM110436  --- | ---  ---  ---  ---  KM110276  KM110277  --- | ---  ---  ---  ---  KM110533  KM110534  KM110535  KM110536  KM110537  KM110538 | ---  ---  ---  ---  KM110610  KM110611  --- |
| Tequital | 5 | CF | 28.27382N  13.98401W | 1 | G33 | Z39 | KM110325 | KM110671* | KM110693 | --- | --- | --- | --- |
| Caldera de la Laguna | 6 | CF | 28.33501N  13.99525W | 3 | G10 | Z228  Z229  Z230a  Z230b | KM110348  KM110349  KM110350 | ---  KM110236  KM110237 | ---  ---  --- | KM110439  KM110440  KM110441  KM110442 | ---  KM110280  KM110281 | ---  KM110542  KM110543 | KM110614  KM110615  KM110616  KM110617 |
|  |  |  |  | 2 | G20 | Z226a  Z226b  Z227 | KM110346  KM110347 | KM110235  KM110678* | ---  KM110700 | KM110437  KM110438 | KM110278  KM110279 | KM110539  KM110540  KM110541 | KM110612  KM110613 |
| Betancuria | 7 | CF | 28.41644N  14.05922W | 2 | G15 | Z38a  Z38b  Z231a  Z231b | KM110324  KM110351 | KM110670*  KM110238 | KM110692  --- | KM110422  KM110443 | KM110269  KM110282 | KM110593  KM110594  KM110544  KM110545 | KM110597  KM110598  KM110618  KM110619 |
| Valle de Aguas Verdes | 8 | CF | 28.47845N  14.05128W | 4 | G13 | Z232  Z233a  Z233b  Z234a  Z234b | KM110352  KM110353  KM110354 | KM110679*  KM110239  --- | KM110701  ---  --- | KM110444  KM110445  KM110446  KM110447 | KM110283  KM110284  --- | KM110546  KM110547  KM110548  --- | ---  KM110620  KM110621 |
| Caldería de la Roja | 12 | NF | 28.62996N  13.83421W | 5 | G7 | Z245a  Z245b  Z246a  Z246b  Z247a  Z247b  Z248a  Z248b  Z306a  Z306b | KM110365  KM110366  KM110367  KM110368  KM110418 | KM110682  ---  ---  KM110242  --- | KM110704  ---  ---  ---  --- | KM110464  KM110465  KM110466  KM110467  KM110468  KM110469  KM110470  KM110520  KM110521 | KM110289  ---  ---  KM110290  --- | KM110555  ---  ---  KM110556  KM110557  --- | KM110634  KM110635  KM110636  KM110637  KM110638  KM110639  --- |
| Corralejo | 10 | NF | 28.72216N  13.8795W | 4 | G8 | Z242  Z243a  Z243b  Z244 | KM110362  KM110363  KM110364 | KM110241  ---  --- | ---  ---  --- | KM110460  KM110461  KM110462  KM110463 | KM110288  ---  --- | KM110554  ---  --- | KM110631  KM110632  KM110633 |
| Villaverde | 11 | NF | 28.65061N  13.91518W | 1 | G8 | Z236a  Z236b | KM110356 | KM110680 | KM110702 | KM110450 | KM110285 | KM110549  KM110550 | KM110624 |
| Faro Toscón | 9 | NF | 28.70336N  14.00779W | 5 | G9 | Z237a  Z237b  Z238a  Z238b  Z239a  Z239b  Z240a  Z240b  Z241a  Z241b | KM110357  KM110358  KM110359  KM110360  KM110361 | KM110681  ---  ---  KM110240  --- | KM110703  ---  ---  ---  --- | KM110451  KM110452  KM110453  KM110454  KM110455  KM110456  KM110457  KM110458  KM110459 | KM110286  KM110287  ---  ---  --- | KM110551  KM110552  KM110553  ---  ---  --- | KM110625  KM110626  KM110627  KM110628  KM110629  KM110630 |
| Salinas de Janubio | 13 | SWL | 28.94288N  13.81886W | 3 | G12 | Z249a  Z249b  Z250a  Z250b  Z251a  Z251b | KM110369  KM110370  KM110371 | ---  KM110243  KM110244 | ---  ---  --- | KM110471  KM110472  KM110473 | ---  KM110291  KM110292 | ---  KM110558  KM110559  KM110560 | KM110640  KM110641  KM110642  KM110643  KM110644  KM110645 |
| Tinajo | 14 | SWL | 29.0614N  13.6777W | 4 | G21 | Z276a  Z276b  Z278a  Z278b | KM110391  KM110392 | KM110253  KM110254 | ---  --- | KM110492  KM110493 | KM110301  KM110302  KM110303 | KM110574  KM110575  KM110576  KM110577 | KM110665  KM110666 |
| Montaña de Tinache, Tinajo | 15 | SWL |  |  | G22 | Z193  Z194 | KM110329  KM110330 | KM110673*  KM110229 | KM110695  --- | ---  --- | ---  --- | ---  --- | ---  --- |
| Tejía | 22 | NEL | 29.02955N  13.51675W | 2 | G11 | Z271  Z272 | KM110386  KM110387 | KM110683*  KM110251 | KM110705  --- | KM110487  KM110488 | KM110297  KM110298 | KM110568  KM110569 | KM110659  KM110660 |
| Barranco Hondo del Valle | 21 | NEL | 29.14139N  13.48203W | 3 | G26 | Z197  Z268 | KM110331  KM110385 | ---  KM110250 | ---  --- | ---  --- | ---  --- | ---  --- | ---  --- |
|  |  |  |  |  | G29 | Z266 | KM110384 | KM110249 | --- | --- | --- | --- | --- |
| Valle de Malpaso | 16 | NEL | 29.1128N  13.51668W | 5 | G23 | Z252  Z253a  Z253b  Z254a  Z254b  Z255  Z256 | KM110372  KM110373  KM110374  KM110375  KM110376 | KM110245  ---  KM110246  ---  --- | ---  ---  ---  ---  --- | KM110474  KM110475  KM110476  KM110477  KM110478 | KM110293  ---  KM110294  ---  --- | KM110561  ---  KM110562  KM110563  ---  --- | KM110646  KM110647  KM110648  ---  KM110649  KM110650 |
| Valle de Guinate | 17 | NEL | 29.17536N  13.50576W | 3 | G25 | Z257  Z259a  Z259b  Z260a  Z260b | KM110377  KM110378  KM110379 | ---  KM110247  KM110248 | ---  ---  --- | KM110479  KM110480  KM110481 | ---  KM110295  KM110296 | ---  KM110564  KM110565  KM110566  KM110567 | KM110651  ---  KM110652  KM110653 |
| Mirador del Río | 20 | NEL | 29.21336N  1.48111W | 7 | G24 | Z262  Z263  Z264  Z265a  Z265b | KM110380  KM110381  KM110382  KM110383 | ---  ---  ---  --- | ---  ---  ---  --- | KM110482  KM110483  KM110484  KM110485  KM110486 | ---  ---  ---  --- | ---  ---  ---  --- | KM110654  KM110655  KM110656  KM110657  KM110658 |
| Montaña de Mojón, La Graciosa | 18 | NEL | 29.2472N  13.518W | 7 | G24 | Z273a  Z273b | KM110388 | KM110684* | KM110706 | KM110489 | --- | KM110570  KM110571 | KM110661 |
| El Vallichuelo, La Graciosa | 19 | NEL | 29.26577N  13.48683W | 7 | G24 | Z274a  Z274b  Z275a  Z275b | KM110389  KM110390 | ---  KM110252 | ---  --- | KM110490  KM110491 | KM110299  KM110300 | ---  KM110572  KM110573 | KM110662  KM110663  KM110664 |
