# Supplementary material for: Spiders on a Hot Volcanic Roof: Colonisation Pathways and Phylogeography of the Canary Islands Endemic Trap-Door Spider Titanidiops canariensis (Araneae, Idiopidae)
Source: PLoS One. 2014 Dec 10;9(12):e115078. doi: 10.1371/journal.pone.0115078 (PMC4262472; doi:10.1371/journal.pone.0115078)
Supplement: S2 Table — Standard genetic diversity indices, nucleotide diversity (π) and haplotype diversity (H) of the cox1 , EF1γ , H3 and AL1 - Hsp70 genes for each GMYC lineage formed by two or more individuals for the entire Titanidiops dataset, T. canariensis , T. maroccanus and the JSF and A clades. (DOC) [file pone.0115078.s004.doc]

| **Group** | **Locus** | **N_ind_** | **N_seq_** | **Pi ± SD** | **H** | **Hd ± SD** |
| --- | --- | --- | --- | --- | --- | --- |
| Overall  *Titanidiops* samples | *cox1* | 96 | 96 | 0.11848 ± 0.00258 | 47 | 0.980 ± 0.004 |
|  | *16S+tRNALeu* | 60 | 60 | 0.07353 ± 0.00306 | 30 | 0.979 ± 0.007 |
|  | *nad1* | 20 | 20 | 0.12501 ± 0.01064 | 18 | 0.984 ± 0.024 |
|  | *EF1γ* | 100 | 113 | 0.00668 ± 0.00044 | 44 | 0.941 ± 0.012 |
|  | *28S* | 53 | 56 | 0.00565 ± 0.00054 | 15 | 0.837 ± 0.032 |
|  | *H3* | 56 | 89 | 0.00818 ± 0.00082 | 26 | 0.839 ± 0.030 |
| Overall  *Titanidiops* *canariensis* | *cox1* | 72 | 72 | 0.10317 ± 0.00187 | 33 | 0.971 ± 0.007 |
|  | *16S+tRNALeu* | 43 | 43 | 0.07353 ± 0.00312 | 30 | 0.979 ± 0.011 |
|  | *nad1* | 17 | 17 | 0.10809 ± 0.00982 | 15 | 0.978 ± 0.031 |
|  | *EF1γ* | 65 | 82 | 0.00298 ± 0.00023 | 23 | 0.896 ± 0.021 |
|  | *28S* | 38 | 39 | 0.00181 ± 0.00029 | 6 | 0.684 ± 0.047 |
|  | *H3* | 41 | 64 | 0.00465 ± 0.00063 | 11 | 0.721 ± 0.041 |
|  | *Hsp70* | 56 | 70 | 0.01285 ± 0.00095 | 38 | 0.957 ± 0.013 |
| Overall  *Titanidiops* *maroccanus* | *cox1* | 24 | 24 | 0.11129 ± 0.00473 | 16 | 0.957 ± 0.025 |
|  | *16S+tRNALeu* | 17 | 17 | 0.06566 ± 0.00487 | 11 | 0.912 ± 0.056 |
|  | *nad1* | 3 | 3 | 0.15754 ± 0.04306 | 3 | 1.000 ± 0.272 |
|  | *EF1γ* | 25 | 31 | 0.00527 ± 0.00048 | 24 | 0.976 ± 0.016 |
|  | *28S* | 15 | 17 | 0.00313 ± 0.00049 | 10 | 0.897 ± 0.056 |
|  | *H3* | 15 | 25 | 0.02246 ± 0.00196 | 22 | 0.990 ± 0.014 |
| G1 | *cox1* | 5 | 5 | 0.00149 ± 0.00052 | 3 | 0.700 ± 0.218 |
|  | *EF1γ* | 5 | 8 | 0.00146 ± 0.00042 | 5 | 0.786 ± 0.151 |
|  | *H3* | 2 | 3 | 0.01458 ± 0.00524 | 3 | 1.000 ± 0.272 |
| G2 | *cox1* | 3 | 3 | 0.00102 ± 0.00048 | 2 | 0.667 ± 0.314 |
|  | *EF1γ* | 3 | 4 | 0.00306 ± 0.00094 | 4 | 1.000 ± 0.177 |
|  | *H3* | 2 | 4 | 0.02875 ± 0.00798 | 4 | 1.000 ± 0.177 |
| G3 | *cox1* | 2 | 2 | 0 | 1 | 0 |
|  | *EF1γ* | 2 | 2 | 0.00376 ± 0.00188 | 2 | 1.000 ± 0.500 |
|  | *H3* | 2 | 3 | 0.03128 ± 0.01083 | 3 | 1.000 ± 0.272 |
| G4 | *cox1* | 3 | 3 | 0.00346 ± 0.00122 | 3 | 1.000 ± 0.272 |
|  | *EF1γ* | 3 | 5 | 0.00376 ± 0.00114 | 5 | 1.000 ± 0.126 |
|  | *H3* | 2 | 4 | 0.02875 ± 0.00761 | 4 | 1.000 ± 0.177 |
| G5 | *cox1* | 5 | 5 | 0.00060 ± 0.00036 | 2 | 0.400 ± 0.237 |
|  | *EF1γ* | 5 | 5 | 0.00075 ± 0.00022 | 2 | 0.600 ± 0.175 |
|  | *H3* | 2 | 3 | 0.03899 ± 0.01191 | 3 | 1.000 ± 0.272 |
| G6 | *cox1* | 3 | 3 | 0.00303 ± 0.00143 | 2 | 0.667 ± 0.314 |
|  | *EF1γ* | 3 | 3 | 0.00326 ± 0.00094 | 3 | 1.000 ± 0.272 |
|  | *H3* | 2 | 2 | 0.01163 ± 0.00581 | 2 | 1.000 ± 0.500 |
| G7 | *cox1* | 5 | 5 | 0.00119 ± 0.00045 | 3 | 0.700 ± 0.218 |
|  | *EF1γ* | 5 | 9 | 0.00137 ± 0.00016 | 4 | 0.806 ± 0.089 |
|  | *H3* | 2 | 3 | 0.00580 ± 0.00158 | 3 | 1.000 ± 0.272 |
|  | *Hsp70* | 4 | 6 | 0.00515 ± 0.00332 | 2 | 0.333 ± 0.215 |
| G8 | *cox1* | 4 | 4 | 0.00077 ± 0.00041 | 2 | 0.500 ± 0.265 |
|  | *EF1γ* | 4 | 5 | 0.00293 ± 0.00071 | 3 | 0.800 ± 0.164 |
|  | *H3* | 2 | 3 | 0.00585 ± 0.00205 | 3 | 1.000 ± 0.272 |
|  | *Hsp70* | 4 | 4 | 0 | 1 | 0 |
| G9 | *cox1* | 5 | 5 | 0 | 1 | 0 |
|  | *EF1γ* | 5 | 9 | 0.00208 ± 0.00047 | 6 | 0.889 ± 0.091 |
|  | *H3* | 2 | 3 | 0.00388 ± 0.00183 | 2 | 0.667 ± 0.314 |
|  | *Hsp70* | 5 | 6 | 0.00640 ± 0.00207 | 2 | 0.533 ± 0.172 |
| G10 | *cox1* | 3 | 3 | 0.00099 ± 0.00047 | 2 | 0.667 ± 0.314 |
|  | *EF1γ* | 3 | 4 | 0.00210 ± 0.00056 | 4 | 1.000 ± 0.177 |
|  | *H3* | 2 | 2 | 0 | 1 | 0 |
|  | *Hsp70* | 3 | 4 | 0.00348 ± 0.00094 | 4 | 1.000 ± 0.177 |
| G11 | *cox1* | 2 | 2 | 0.00446 ± 0.00223 | 2 | 1.000 ± 0.500 |
|  | *EF1γ* | 2 | 2 | 0 | 1 | 0 |
|  | *H3* | 2 | 2 | 0 | 1 | 0 |
|  | *Hsp70* | 2 | 2 | 0.00168 ± 0.00084 | 2 | 1.000 ± 0.500 |
| G12 | *cox1* | 3 | 3 | 0 | 1 | 0 |
|  | *EF1γ* | 3 | 3 | 0 | 1 | 0 |
|  | *H3* | 3 | 3 | 0.00220 ± 0.00104 | 2 | 0.667 ± 0.314 |
|  | *Hsp70* | 3 | 6 | 0.00981 ± 0.00255 | 5 | 0.933 ± 0.122 |
| G13 | *cox1* | 4 | 4 | 0 | 1 | 0 |
|  | *EF1γ* | 4 | 6 | 0.00171 ± 0.00054 | 3 | 0.800 ± 0.172 |
|  | *H3* | 2 | 3 | 0.00192 ± 0.00091 | 2 | 0.667± 0.314 |
|  | *Hsp70* | 4 | 5 | 0.00842 ± 0.00197 | 4 | 0.900 ± 0.161 |
| G14 | *cox1* | 2 | 2 | 0.00594 ± 0.00297 | 2 | 1.000 ± 0.500 |
|  | *EF1γ* | 2 | 3 | 0.00084 ± 0.00040 | 2 | 0.667 ± 0.314 |
| G15 | *cox1* | 2 | 2 | 0.00149 ± 0.00074 | 2 | 1.000 ± 0.500 |
|  | *EF1γ* | 2 | 2 | 0 | 1 | 0 |
|  | *H3* | 2 | 4 | 0.02216 ± 0.00824 | 4 | 1.000 ± 0.177 |
|  | *Hsp70* | 2 | 3 | 0.01433 ± 0.00581 | 3 | 1.000 ± 0.272 |
| G16 | *cox1* | 5 | 5 | 0.00297 ± 0.00062 | 4 | 0.900 ± 0.161 |
|  | *EF1γ* | 5 | 5 | 0.00146 ± 0.00059 | 3 | 0.700 ± 0.218 |
|  | *H3* | 2 | 3 | 0.03488 ± 0.01473 | 3 | 1.000 ± 0.272 |
|  | *Hsp70* | 5 | 6 | 0.01404 ± 0.00208 | 5 | 0.933 ± 0.122 |
| G17 | *cox1* | 3 | 3 | 0 | 1 | 0 |
|  | *EF1γ* | 3 | 3 | 0.00082 ± 0.00039 | 2 | 0.667 ± 0.314 |
|  | *H3* | 2 | 3 | 0.03661 ± 0.01392 | 3 | 1.000 ± 0.272 |
|  | *Hsp70* | 3 | 3 | 0.00771 ± 0.00364 | 2 | 0.667 ± 0.314 |
| G18 | *cox1* | 2 | 2 | 0 | 2 | 0 |
|  | *EF1γ* | 2 | 2 | 0 | 1 | 0 |
|  | *H3* | 2 | 3 | 0.00601 ± 0.00211 | 3 | 1.000 ± 0.272 |
|  | *Hsp70* | 2 | 2 | 0.00360 ± 0.00180 | 2 | 1.000 ± 0.500 |
| G19 | *cox1* | 6 | 6 | 0 | 1 | 0 |
|  | *EF1γ* | 3 | 5 | 0.00174 ± 0.00048 | 4 | 0.900 ± 0.161 |
|  | *H3* | 3 | 6 | 0.03168 ± 0.00625 | 6 | 1.000 ± 0.096 |
|  | *Hsp70* | 2 | 2 | 0 | 1 | 0 |
| G20 | *cox1* | 2 | 2 | 0 | 2 | 0 |
|  | *EF1γ* | 2 | 2 | 0 | 1 | 0 |
|  | *H3* | 2 | 3 | 0.01198 ± 0.00565 | 2 | 0.667 ± 0.314 |
|  | *Hsp70* | 2 | 2 | 0.00176 ± 0.00088 | 2 | 1.000 ± 0.500 |
| G21 | *cox1* | 2 | 2 | 0 | 1 | 0 |
|  | *EF1γ* | 2 | 2 | 0.00123 ± 0.00062 | 2 | 1.000 ± 0.500 |
|  | *H3* | 2 | 4 | 0.00192 ± 0.00059 | 2 | 0.667 ± 0.204 |
|  | *Hsp70* | 2 | 2 | 0 | 1 | 0 |
| G22 | *cox1* | 2 | 2 | 0.00902 ± 0.00451 | 2 | 1.000 ± 0.500 |
| G23 | *cox1* | 5 | 5 | 0 | 1 | 0 |
|  | *EF1γ* | 5 | 5 | 0.00075 ± 0.00022 | 2 | 0.600 ± 0.175 |
|  | *H3* | 2 | 3 | 0.00440 ± 0.00147 | 3 | 1.000 ± 0.272 |
|  | *Hsp70* | 4 | 5 | 0.01136 ± 0.00330 | 5 | 1.000 ± 0.126 |
| G24 | *cox1* | 7 | 7 | 0.00340 ± 0.00089 | 3 | 0.667 ± 0.160 |
|  | *EF1γ* | 7 | 8 | 0.00138 ± 0.00038 | 5 | 0.786 ± 0.151 |
|  | *H3* | 2 | 4 | 0.00434 ± 0.00158 | 3 | 0.833 ± 0.222 |
|  | *Hsp70* | 7 | 9 | 0.00761 ± 0.00125 | 7 | 0.889 ± 0.091 |
| G25 | *cox1* | 3 | 3 | 0.00101 ± 0.00047 | 2 | 0.667 ± 0.314 |
|  | *EF1γ* | 3 | 3 | 0.00246 ± 0.00086 | 3 | 1.000 ± 0.272 |
|  | *H3* | 2 | 4 | 0.00528 ± 0.00127 | 4 | 1.000 ± 0.177 |
|  | *Hsp70* | 2 | 3 | 0.00790 ± 0.00219 | 3 | 1.000± 0.272 |
| G26 | *cox1* | 2 | 2 | 0.00625 ± 0.00313 | 2 | 1.000 ± 0.500 |
|  | *H3* | 2 | 2 | 0.00288 ± 0.00144 | 2 | 1.000 ± 0.500 |
| *Titanidiops* *canariensis*  clade JSF | *cox1* | 20 | 20 | 0.03854 ± 0.00530 | 10 | 0.889 ± 0.051 |
|  | *16S+tRNALeu* | 15 | 15 | 0.01995 ± 0.00213 | 12 | 0.962 ± 0.040 |
|  | *nad1* | 8 | 8 | 0.04987 ± 0.00732 | 6 | 0.893 ± 0.111 |
|  | *EF1γ* | 17 | 19 | 0.00201 ± 0.00029 | 6 | 0.795 ± 0.060 |
|  | *28S* | 12 | 12 | 0.00079 ± 0.00017 | 2 | 0.485 ± 0.106 |
|  | *H3* | 13 | 22 | 0.00655 ± 0.00042 | 11 | 0.853 ± 0.063 |
|  | *Hsp70* | 14 | 16 | 0.01522 ± 0.00242 | 8 | 0.858 ± 0.063 |
| *Titanidiops* *canariensis*  clade A | *cox1* | 43 | 43 | 0.07935 ± 0.00286 | 19 | 0.950 ± 0.013 |
|  | *16S+tRNALeu* | 23 | 23 | 0.07338 ± 0.00467 | 18 | 0.980 ± 0.018 |
|  | *nad1* | 6 | 6 | 0.11581 ± 0.02510 | 6 | 1.000 ± 0.096 |
|  | *EF1γ* | 43 | 56 | 0.00259 ± 0.00019 | 19 | 0.916 ± 0.017 |
|  | *28S* | 20 | 21 | 0.00193 ± 0.00057 | 7 | 0.595 ± 0.108 |
|  | *H3* | 18 | 29 | 0.00468 ± 0.00042 | 7 | 0.828 ± 0.037 |
|  | *Hsp70* | 40 | 52 | 0.01094 ± 0.00071 | 29 | 0.939 ± 0.022 |
